# Novel Gut Microbiota Patterns Involved in the Attenuation of Dextran Sodium Sulfate-Induced Mouse Colitis Mediated by Glycerol Monolaurate via Inducing Anti-inflammatory Responses

**DOI:** 10.1128/mBio.02148-21

**Published:** 2021-10-12

**Authors:** Qiufen Mo, Tao Liu, Aikun Fu, Shengyue Ruan, Hao Zhong, Jun Tang, Minjie Zhao, Yang Li, Songming Zhu, Haiying Cai, Fengqin Feng

**Affiliations:** a College of Biosystems Engineering and Food Science, National Engineering Laboratory of Intelligent Food Technology and Equipment, Key Laboratory for Agro-Products Postharvest Handling of Ministry of Agriculture and Rural Affairs, Key Laboratory for Agro-Products Nutritional Evaluation of Ministry of Agriculture, Zhejiang Key Laboratory for Agro-Food Processing, Zhejiang University, Hangzhou, Zhejiang, China; b Ningbo Institute of Zhejiang University, Ningbo, Zhejiang, China; c Institute of Biology, Westlake Institute for Advanced Study, Westlake University, Hangzhou, Zhejiang, China; d School of Biological and Chemical Engineering, Zhejiang University of Science and Technology, Hangzhou, Zhejiang, China; Cornell University

**Keywords:** IBD, glycerol monolaurate, microbiota pattern, *Bifidobacterium*, *Helicobacter*, Tregs, bifidobacterium, inflammatory bowel disease

## Abstract

Inflammatory bowel disease (IBD) is a type of immune-mediated chronic and relapsing inflammatory gastrointestinal symptoms. IBD cannot be completely cured because of the complex pathogenesis. Glycerol monolaurate (GML), naturally found in breast milk and coconut oil, has excellent antimicrobial, anti-inflammatory, and immunoregulatory functions. Here, the protective effect of GML on dextran sodium sulfate (DSS)-induced mouse colitis and the underlying gut microbiota-dependent mechanism were assessed in C57BL/6 mice pretreated or cotreated with GML and in antibiotic-treated mice transplanted with GML-modulated microbiota. Results showed that GML pretreatment has an advantage over GML cotreatment in alleviating weight loss and reducing disease activity index (DAI), colonic histological scores, and proinflammatory responses. Moreover, the amounts of *Lactobacillus* and *Bifidobacterium* and fecal propionic acid and butyric acid were elevated only in mice pretreated with GML upon DSS induction. Of note, fecal microbiota transplantation (FMT) from GML-pretreated mice achieved faster and more significant remission of DSS-induced colitis, manifested as reduced DAI, longer colon, decreased histological scores, and enhanced colonic Foxp3^+^ regulatory T cells (Tregs) and ratio of serum anti-inflammatory/proinflammatory cytokines, as well as the reconstruction of microbial communities, including elevated Helicobacter ganmani and decreased pathogenic microbes. In conclusion, GML-mediated enhancement of *Bifidobacterium* and fecal short-chain fatty acids (SCFAs) could be responsible for the anticolitis effect. FMT assay confirmed that gut microbiota modulated by GML was more resistant to DSS-induced colitis via elevating beneficial *H. ganmani* and establishing Treg tolerant phenotype. Importantly, colitis remission induced by GML is associated with novel gut microbiota patterns, even though different microbial contexts were involved.

## INTRODUCTION

The rising incidence of inflammatory bowel disease (IBD) worldwide, represented by ulcerative colitis and Crohn’s disease, coincides with a marked change of environmental factors (1–3). Recent advances in the science of microbiome and IBD have highlighted that the gut microbiota which inhabits the gastrointestinal tract and can be highly and dynamically affected by dietary components is closely related to the IBD pathogenesis (4). Progress in next-generation sequencing technology has documented lower microbial diversity, changes in the gut microbial profiles (such as decreased *Firmicutes*, *Clostridia* XIVa, and *Bifidobacterium* and increased *Enterobacteriaceae*, Proteus, and Escherichia coli), and lower short-chain fatty acid (SCFA) contents in IBD patients relative to healthy individuals, which is referred to as dysbiosis (5, 6). It is widely accepted that microbial dysbiosis could affect the balance between regulatory T cells (Foxp3^+^ Tregs) and T helper 17 cells (Th17) in IBD (7). Tregs were demonstrated to maintain intestinal homeostasis by secreting anti-inflammatory cytokines, mainly interleukin-10 (IL-10), and inversely regulating Th17 (8), which is increased in the colonic mucosa and serum of IBD patients (9). The chronic, nonspecific, and relapsing immune-mediated inflammation has been described as one of the leading causes of IBD (10) and seems to impact the gut microbiota (11), which in turn facilitates the aggravation of inflammatory response (7). The cytokine profiles seen in IBD patients were characterized by elevated tumor necrosis factor alpha (TNF-α), IL-6, and IL-23, etc., but modest production of IL-10 (12). This cytokine disequilibrium is profoundly involved in the selective colonization by microbial taxa (13). Although such causality remains obscure, microbial dysbiosis is often accompanied by inflammation development and may act synergistically to cause IBD, which cannot be completely cured and requires medication, including antibiotics (ABX), immunomodulators, and biologics to extend the remission period (14). However, medical treatment of IBD requires chronic application of drugs that may increase the risk of adverse effects (5, 15). Based on the above, rational alternative approaches targeting the abnormal microbiota and inflammation through bioactive dietary components are attracting increased attention (15).

Glycerol monolaurate (GML), naturally found in human milk and coconut oil, is a generally recognized as safe food emulsifier with distinguished antimicrobial, antiviral, and anti-inflammatory properties (16). GML not only has been extensively used in food and cosmetics (17, 18) but also has been commonly applied to inhibit potential bacterial pathogens (19) and prevent simian immunodeficiency virus (SIV) infection and virally induced vaginal inflammation (16). Moreover, GML has been demonstrated to possess immunomodulatory functions as evidenced by suppressing human T cell functions and signaling and regulating cytokine production (20). Other research speculated that oral administration of GML could restrain excessive inflammatory responses in IBD because of its immunomodulatory functions (21). Recently, GML has been assessed as one of the most promising pharmacological candidate compounds (16). Our previous studies demonstrated that GML (1,600 mg kg^−1^) could significantly upregulate the concentrations of anti-inflammatory cytokines and relative abundances of beneficial indigenous microbiota (especially *Clostridia* XIVa) in normal mice, both of which were implicated in intestinal microecological balance (22). Furthermore, 1,600 mg kg^−1^ GML has the potential to improve lipid metabolism and ameliorates related low-grade inflammation in high-fat-diet (HFD)-fed mice by modulating the gut microbiota composition (23). GML has pleiotropic characteristics that make it an attractive anti-inflammatory agent. However, the effect of GML on experimental colitis model or IBD patients has not been sufficiently elucidated. Here, we investigate the beneficial anticolitis effect of GML in dextran sulfate sodium (DSS)-induced colitis in C57BL/6 mice and elucidate the important aspect of mechanisms involved.

## RESULTS

### GML pretreatment prevents DSS-induced acute colitis.

**FIG 1:**
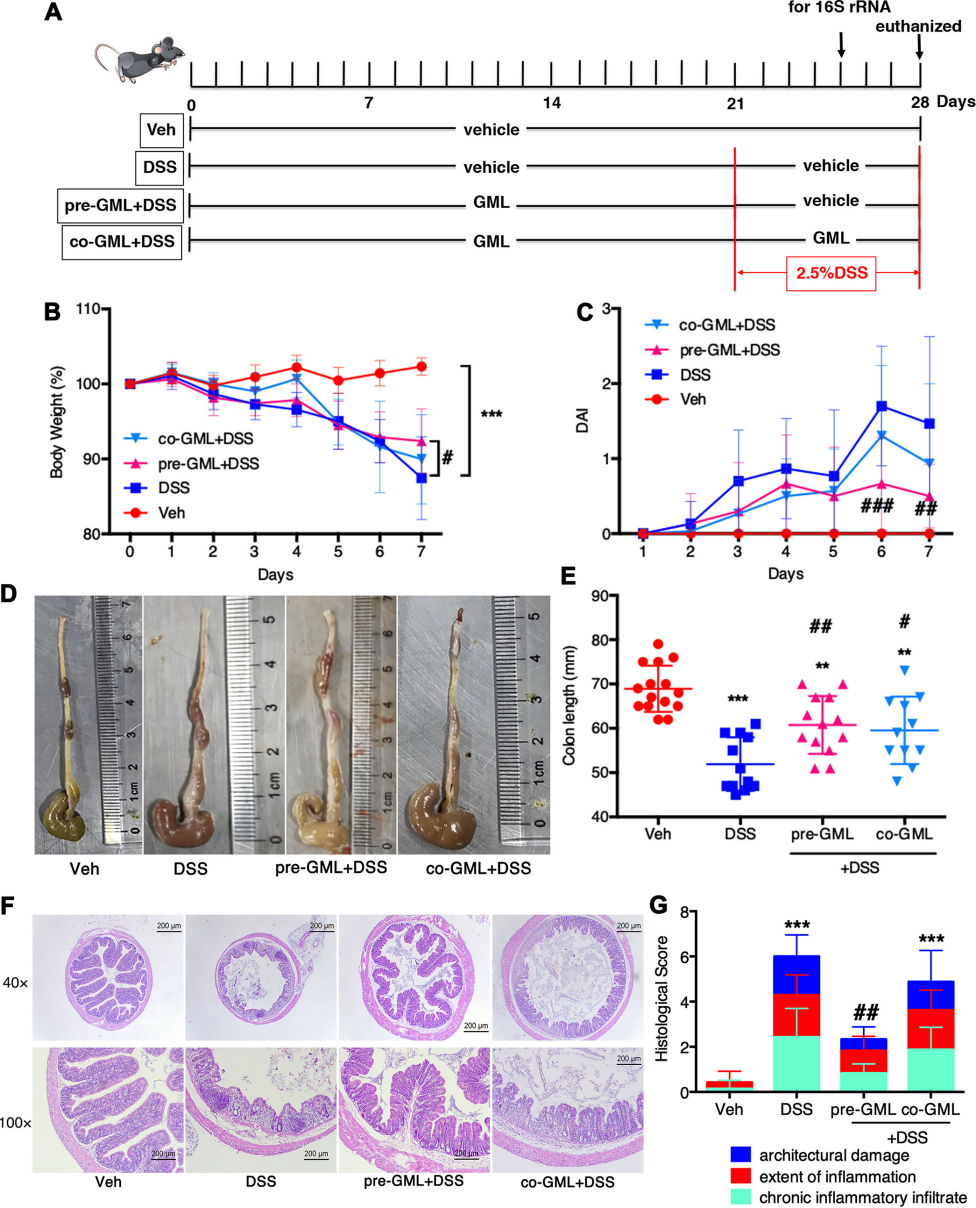
GML pretreatment prevents DSS-induced acute colitis. (A) The schematic diagram of experimental design and procedure. (B) Body weight changes. (C) The disease activity index (DAI) assessment. (D and E) Colon length at day 7. (F and G) H&E staining analysis (F) and histology score (G) of colonic tissues. Each dot represents an individual mouse. (B to G) *n* = 6 to 15 mice/group. *, *P* < 0.05; **, *P* < 0.01; ***, *P* < 0.001, versus Veh group and #, *P* < 0.05; ##, *P* < 0.01, versus DSS group, determined by a one-way ANOVA followed by Bonferroni’s *post hoc* test.

In the pilot experiments, dose-response curves of DSS and GML were performed. DSS (2.5%) and GML (6 mg/mouse/day) were considered to be the optimal dosages for formal experiments (see Fig. S1 in the supplemental material). In the present study, DSS resulted in severe diarrhea and obvious weight loss (Fig. 1B), reduction in the colon length (Fig. 1D and E), and histological signs of experimental colitis, including colonic epithelial damage and chronic inflammatory infiltrate (Fig. 1F and G). Of note, GML pretreatment improved the body weight significantly at day 7 post-DSS application in comparison to the DSS group, whereas GML cotreatment led to less weight loss at the earlier 4-day point but not at days 5 to 7 compared to the DSS group (Fig. 1B). There was 80% survival in mice from DSS and co-GML+DSS groups, while GML pretreatment improved survival to 100% upon DSS induction (Fig. S2). The disease activity index (DAI) remained null for the vehicle (Veh) group and was remarkably lower in GML-pretreated mice than in mice in the DSS group (*P* < 0.001 at day 6 and *P* < 0.01 at day 7) (Fig. 1C). The shortening of average colon length in mice of the DSS group (53.7 ± 5.64 mm) was significantly reversed in GML-pretreated mice (63.7 ± 4.94 mm; *P* < 0.01) and GML-cotreated mice (62.5 ± 6.37 mm; *P* < 0.05) (Fig. 1D and E). Moreover, blind histological assessment of colonic micrographs revealed that pretreatment with GML significantly reversed DSS-induced epithelial damage, chronic inflammatory infiltrate, and the extent of inflammation, resulting in a large decrease of overall histological score (*P* < 0.01). However, cotreatment with GML did not improve the histological parameters of colitis (Fig. 1F and G).

10.1128/mBio.02148-21.1 FIG S1 Dose-response curve of DSS and GML. (A) Effect of different doses of DSS on the percentage of body weight in mice. (B) Effects of different doses of GML on the percentage of body weight in DSS-induced colitis mice. (C) Effects of different doses of GML on the disease activity index in DSS-induced colitis mice. *, *P* < 0.05; **, *P* < 0.01, versus DSS group, determined by a one-way ANOVA followed by Bonferroni’s *post hoc* test. Download FIG S1, TIF file, 0.2 MB. Copyright © 2021 Mo et al. 2021 Mo et al. https://creativecommons.org/licenses/by/4.0/ This content is distributed under the terms of the Creative Commons Attribution 4.0 International license.

10.1128/mBio.02148-21.2 FIG S2 Survival curve of the four groups of mice with or without DSS treatment. Download FIG S2, TIF file, 0.07 MB. Copyright © 2021 Mo et al. 2021 Mo et al. https://creativecommons.org/licenses/by/4.0/ This content is distributed under the terms of the Creative Commons Attribution 4.0 International license.

### GML pretreatment reduced DSS-induced proinflammatory status.

**FIG 2:**
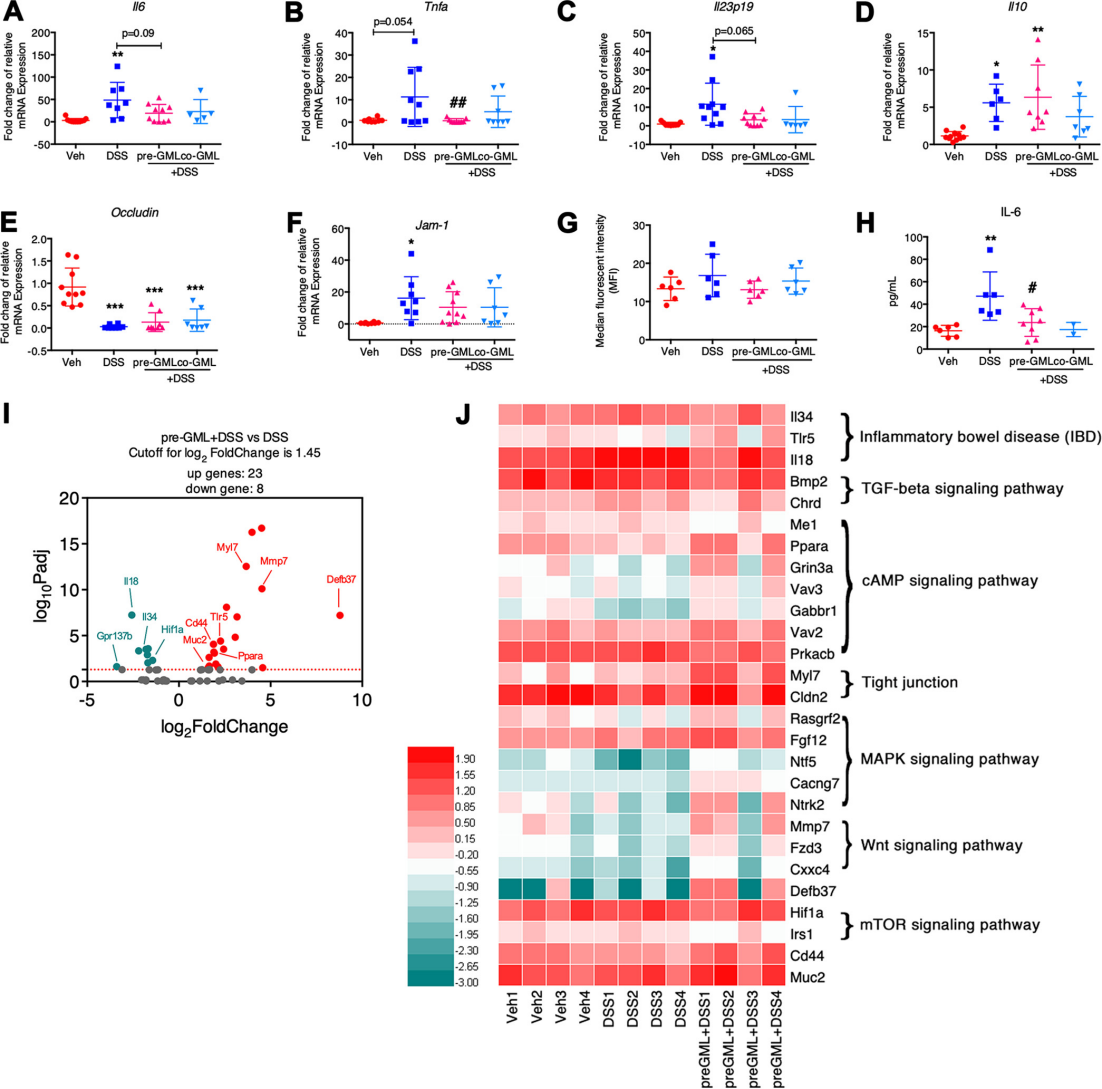
GML pretreatment reduced DSS-induced proinflammatory status. (A to F) Colonic mRNA levels of *Il6* (A), *Tnfa* (B), *Il23p19* (C), *Il10* (D), *Occludin* (E), and *Jam-1* (F) assessed by RT-qPCR. (G) *In vivo* intestinal permeability characterized as median fluorescence intensity (MFI) of FITC-dextran. (H) Serum levels of IL-6 assessed by enzyme-linked immunosorbent assay (ELISA). (I and J) Volcano plot of differentially expressed genes (DEGs) (I) and heatmap representation of DEG levels from colon tissue between pre-GML+DSS and DSS groups as measured by RNA-Seq technology (J). Twenty-three genes were upregulated and 8 genes were downregulated (log_2_|fold change| ≥ 1.45, adjusted *P* < 0.05) between pre-GML+DSS and DSS groups. Expression level was represented as each gene’s fragments per kilobase million (FPKM) value. *, *P < *0.05; **, *P* < 0.01; ***, *P* < 0.001, versus Veh group and #, *P* < 0.05, ##, *P* < 0.01, versus DSS group for panels A to H, determined by a one-way ANOVA followed by Bonferroni’s *post hoc* test.

To illuminate the underlying mechanisms of how GML remitted the colitis severity, we quantified the expression of pro- and anti-inflammatory cytokines and tight junction proteins (TJPs) in the colonic samples. DSS enhanced the expression of *Il6* (*P* < 0.01), *Tnfa* (*P* = 0.054), *Il23p19* (*P* < 0.01), *Il10* (*P* < 0.05), and *Jam-1* (*P < *0.05) and reduced the expression of *Occludin* (*P* < 0.001), *Il17a* (*P* = 0.098), and *Foxp3* (*P* < 0.01). GML pretreatment suppressed the increase of *Il6* (*P* = 0.09), *Tnfa* (*P* < 0.01), and *Il23p19* (*P* = 0.065), while elevated *Il10* (*P* < 0.01) and *Jam-1* as well as decreased *Occludin* (*P* < 0.001) and *Il17a* (*P* = 0.052) levels were still present (Fig. 2A to F and Fig. S3A). However, the expression of these cytokines, except *Occludin* and *Il17a*, in the co-GML+DSS group remained unchanged relative to the Veh group. Interesting, the intestinal permeability analysis showed no difference in fluorescein isothiocyanate (FITC)-dextran concentration among different groups (Fig. 2G) because of the inconsonant change in the mRNA level of TJPs. In accordance with increased mRNA production, serum IL-6 was elevated dramatically in DSS-treated mice but reduced significantly in mice pretreated with GML (Fig. 2H), whereas colonic production of *Zo1* and *Il22*, as well as serum levels of lipopolysaccharide (LPS), IL-10, IL-22, IL-17, and TNF-α, was not significantly affected by any treatments (Fig. S3A and B). Moreover, colonic transcriptional profiles analyzed using transcriptome sequencing (RNA-Seq) technology demonstrated that the expression levels of inflammation-related genes were markedly different between the DSS and pre-GML+DSS groups (Fig. 2I and J). Results showed that DSS increased the expression of gene *il18* (1.9-fold), which was associated with IBD pathways, while GML pretreatment reversed the increase of *il18* (2.6-fold, Fig. 2J, Fig. S3C, and Table S1). DSS specifically decreased the expression of *Lgr5* (4.7-fold), which was identified as a marker of intestinal stem cells at the base of crypt (24), and genes related to the cAMP pathway including *Gli1* (1.7-fold) and *Hhip* (2.3-fold) but increased the chemokine genes, such as *Ccl7* (2.1-fold) and *Ccl8* (2.5-fold) (Fig. S3C and Table S1). Other genes involved in tight junction, IBD, cAMP, Wnt, and mitogen-activated protein kinase (MAPK) signaling pathways, such as *Myl7* (3.7-fold), *Tlr5* (2.3-fold), *Cd44* (1.9-fold), *Ppara* (1.9-fold), *Gabbr1* (3.2-fold), *Mmp7* (4.5-fold), *Fzd3* (2.4-fold), *Muc2* (1.6-fold), and *Defb37* (8.8-fold), were upregulated, whereas *Il34* (1.8-fold), *Isl1* (1.9-fold), *Gpr137b* (3.4-fold), *Prkacb* (1.7-fold), *Bmp2* (2.2-fold), and *Hif1a* (1.4-fold) were downregulated specifically in the pre-GML+DSS group (Fig. 2J, Fig. S3C, and Table S2). These data indicated that GML moderated DSS-induced experimental colitis by inhibiting the inflammation-related signaling pathways.

10.1128/mBio.02148-21.3 FIG S3 (A) Colonic expression of *Foxp3*, *Il17a*, and *Il22*, as well as tight junction-related genes *Zo1* assessed by RT-qPCR. (B) Serum secretion of LPS, IL-17, TNF-α, IL-10, and IL-22 assessed by ELISA. (C) Heatmap representation of overall hierarchical clustering DEG levels from colon tissue based on the fragments per kilobase million (FPKM) value between pre-GML+DSS and DSS groups as measured by RNA-Seq technology. *, *P* < 0.05, versus Veh group, determined by a one-way ANOVA followed by Bonferroni’s *post hoc* test. Download FIG S3, TIF file, 0.2 MB. Copyright © 2021 Mo et al. 2021 Mo et al. https://creativecommons.org/licenses/by/4.0/ This content is distributed under the terms of the Creative Commons Attribution 4.0 International license.

10.1128/mBio.02148-21.9 TABLE S1 The complete lists for differentially expressed genes (DEGs) among Veh, DSS, and pre-GML+DSS groups. Download Table S1, XLSX file, 0.10 MB. Copyright © 2021 Mo et al. 2021 Mo et al. https://creativecommons.org/licenses/by/4.0/ This content is distributed under the terms of the Creative Commons Attribution 4.0 International license.

10.1128/mBio.02148-21.10 TABLE S2 The identified KEGG pathways involved in differentially expressed genes (DEGs) among Veh, DSS, and pre-GML+DSS groups. Download Table S2, XLSX file, 0.05 MB. Copyright © 2021 Mo et al. 2021 Mo et al. https://creativecommons.org/licenses/by/4.0/ This content is distributed under the terms of the Creative Commons Attribution 4.0 International license.

### GML pretreatment prevents DSS-induced microbial dysbiosis.

**FIG 3:**
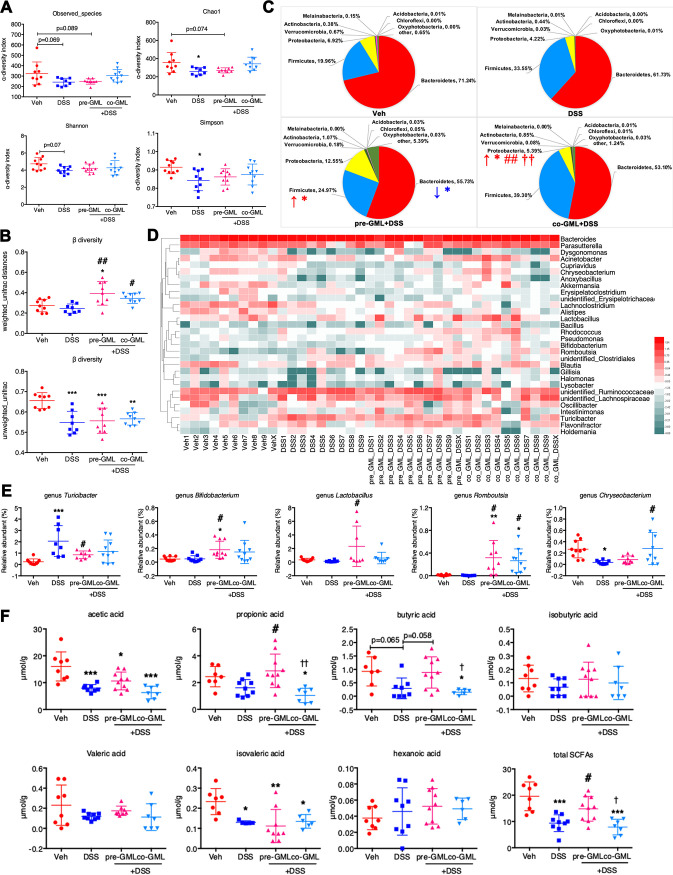
GML pretreatment prevents DSS-induced microbial dysbiosis. (A) α-Diversity of gut microbiota represented by Observed_species, Chao1, Shannon, and Simpson indices. (B) β-Diversity of gut microbiota based on weighted UniFrac distance and unweighted UniFrac distance. (C) Relative abundances of gut microbiota at the phylum level. (D) Heatmap depicting the log_10_|relative abundance| of top 30 microbial genera, when the taxa with the relative abundance as zero were expressed as −4. (E) Relative abundance of five differential microbial genera. (F) The production of microbial metabolite SCFAs from feces among the four different groups. Each dot represents an individual mouse (*n* = 9 to 10 mice/group). *, *P* < 0.05; **, *P* < 0.01; ***, *P* < 0.001, versus Veh group; #, *P* < 0.05; ##, *P* < 0.01, versus DSS group; †, *P* < 0.05; ††, *P* < 0.01, versus pre-GML+DSS group, determined by a nonparametric factorial Kruskal-Wallis test followed by the Mann-Whitney U test when *P* was <0.05.

Previous studies revealed that microbial dysbiosis was observed in IBD. To investigate whether GML could reverse the dysbiosis, we further evaluated the diversity and composition of fecal microbiota in different groups. Compared with the Veh group, microbial community in the DSS group exhibited reduced α-diversity as assessed by Observed_species (*P* = 0.069), Chao1 (*P* < 0.05), Shannon (*P* = 0.07), and Simpson (*P* < 0.05) indices. However, the Shannon and Simpson indices in the pre-GML+DSS and co-GML+DSS groups were similar to those of the Veh group (Fig. 3A). Using the operational taxonomic unit (OTU)-level Bray-Curtis dissimilarity matrix, the permutational multivariate analysis of variance (PERMANOVA) indicated a statistical separation among the four groups (*P* = 0.003; Fig. S4A). The overall difference in the microbial structure (β-diversity) in the pre-GML+DSS (*P* < 0.01) and co-GML+DSS (*P* < 0.05) groups was distinct from that of the DSS group, based on weighted but not unweighted UniFrac distances (Fig. 3B). Referring to the taxonomic shifts, phylum-level decreases in the relative abundance of *Bacteroidetes* and increases in *Firmicutes*, and higher ratios of *Firmicutes*/*Bacteroidetes*, were observed in mice pretreated with GML relative to animals in the Veh group (*P* < 0.05 for all). *Proteobacteria* were more abundant in the co-GML+DSS group than in other groups (Fig. 3C). At the genus level, DSS increased the level of *Turicibacter* and decreased *Chryseobacterium* (Fig. 3D and E), whereas GML pretreatment inhibited the increase of *Turicibacter* but possessed higher percentages of *Romboutsia*, *Lactobacillus*, and *Bifidobacterium* (specifically Bifidobacterium animalis), while GML cotreatment was associated with elevated *Romboutsia* and *Chryseobacterium* (Fig. 3D and E and Fig. S4C and D). Similarly, as shown in Fig. S4B, the linear discriminant analysis (LDA) effect size (LEfSe) algorithm ranked *Erysipelotrichia* (from class to family) and genus *Turicibacter* as the main bacterial taxa of the DSS group, while the Veh group was enriched with family *Muribaculaceae* within *Bacteroidetes*, in contrast with those of the pre-GML+DSS group and co-GML+DSS group, which were dominated by *Firmicutes* and *Proteobacteria*, respectively. Furthermore, GML pretreatment rescued the concentrations of microbe-derived SCFAs. Results showed that the concentrations of butyric acid tended to decrease in the DSS group relative to the Veh group (*P* = 0.065), but mice pretreated with GML exhibited increased butyric acid (*P* = 0.058) and propionic acid (*P* < 0.05) compared to mice in the DSS group. Decreased acetic acid and isovaleric acid and unchanged isobutyric acid, valeric acid, and hexanoic acid were observed in the DSS group compared with the Veh group. Consequently, the total SCFAs were lower in the DSS group than the Veh group (*P* < 0.001) but higher in the pre-GML+DSS than the DSS group (*P* < 0.05). However, a significant reduction in propionic acid, butyric acid, and total SCFAs was observed in the co-GML+DSS group compared with Veh and pre-GML+DSS groups (Fig. 3F). These data indicated that GML-moderated DSS-induced experimental colitis was closely related to the reconstruction of microbial patterns. Since mice in the pre-GML+DSS group were more resistant to DSS-induced colitis than those in the co-GML+DSS group, which in part excluded the direct effect of GML against the colitis, we concluded that the beneficial effects of GML pretreatment could be mediated by the differential microbial taxa, including *Lactobacillus* and *Bifidobacterium*, and the recovery of butyric acid and propionic acid.

10.1128/mBio.02148-21.4 FIG S4 GML pretreatment prevents DSS-induced microbial dysbiosis. (A) 3D PCoA plot based on OTU-level Bray-Curtis dissimilarity matrix and PERMANOVA. (B) Key phylotypes of gut microbiota identified by LEfSe analysis with linear discriminant analysis (LDA) scores of >4 among Veh, DSS, pre-GML+DSS, and co-GML+DSS groups. (C) Heatmap depicting the log_10_|relative abundance| of top 30 microbial species, when the taxa with the relative abundance as zero were expressed as −4. (D) Relative abundance of 10 differential microbial species. Values are presented as means ± SD; *n* = 9 to 10 mice/group. *, *P* < 0.05; **, *P* < 0.01; ***, *P* < 0.001, versus Veh group; #, *P* < 0.05; ##, *P* < 0.01; ###, *P* < 0.001, versus DSS group. †††, *P* < 0.01 versus pre-GML+DSS group, determined by a nonparametric factorial Kruskal-Wallis test followed by the Mann-Whitney U test when *P* was <0.05. Download FIG S4, TIF file, 0.5 MB. Copyright © 2021 Mo et al. 2021 Mo et al. https://creativecommons.org/licenses/by/4.0/ This content is distributed under the terms of the Creative Commons Attribution 4.0 International license.

### FMT from GML-treated donor mice prevented DSS-induced colitis in antibiotic-treated mice.

**FIG 4:**
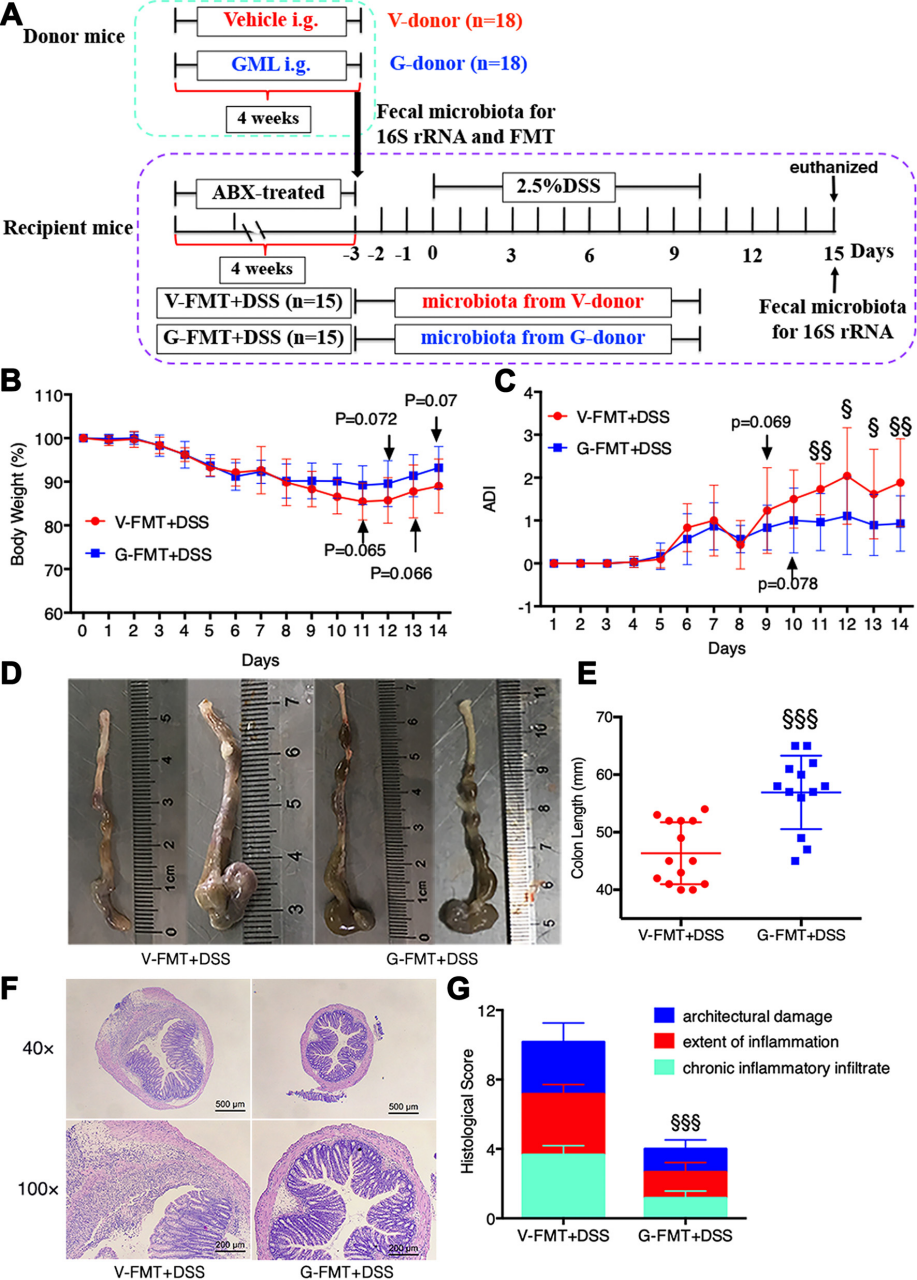
FMT from GML-treated mice prevented DSS-induced colitis in antibiotic-treated mice. (A) The schematic diagram of experimental design and procedure of FMT experiment. (B) Body weight changes. (C) DAI assessment. (D and E) Colon length measurement. (F and G) H&E staining analysis (F) and histology score (G) of colonic tissues. (B to E) *n* = 12 to 15 mice/group; (F and G) *n* = 8 mice/group. §, *P* < 0.05; §§, *P* < 0.01; §§§, *P* < 0.001, versus V-FMT+DSS group, determined by a two-tailed Student *t* test.

Fecal microbiota transplant (FMT) experiments were thus performed in antibiotic-treated mice, which were modeled to colitis by DSS application during the FMT course (Fig. 4A), therefore allowing for a quantifiable effect of GML-modulated microbiota on protection against the colitis-related syndromes. Results showed that FMT from GML-treated donor mice significantly attenuated the DAI (Fig. 4C), colon length shortening (Fig. 4D and E), and histological symptoms and scores (Fig. 4F and G) compared with FMT from vehicle-treated mice. It should be mentioned that FMT from vehicle-treated mice contained a healthy microbial community as well, which may illustrate the undifferentiated weight between the G-FMT+DSS and V-FMT+DSS groups during the colitis period. In contrast, a tendency higher in the body weight percentage was maintained in the G-FMT+DSS group relative to the V-FMT+DSS group at days 11 to 14 (Fig. 4B).

### FMT from GML-treated mice promoted anti-inflammatory response upon colitis induction.

**FIG 5:**
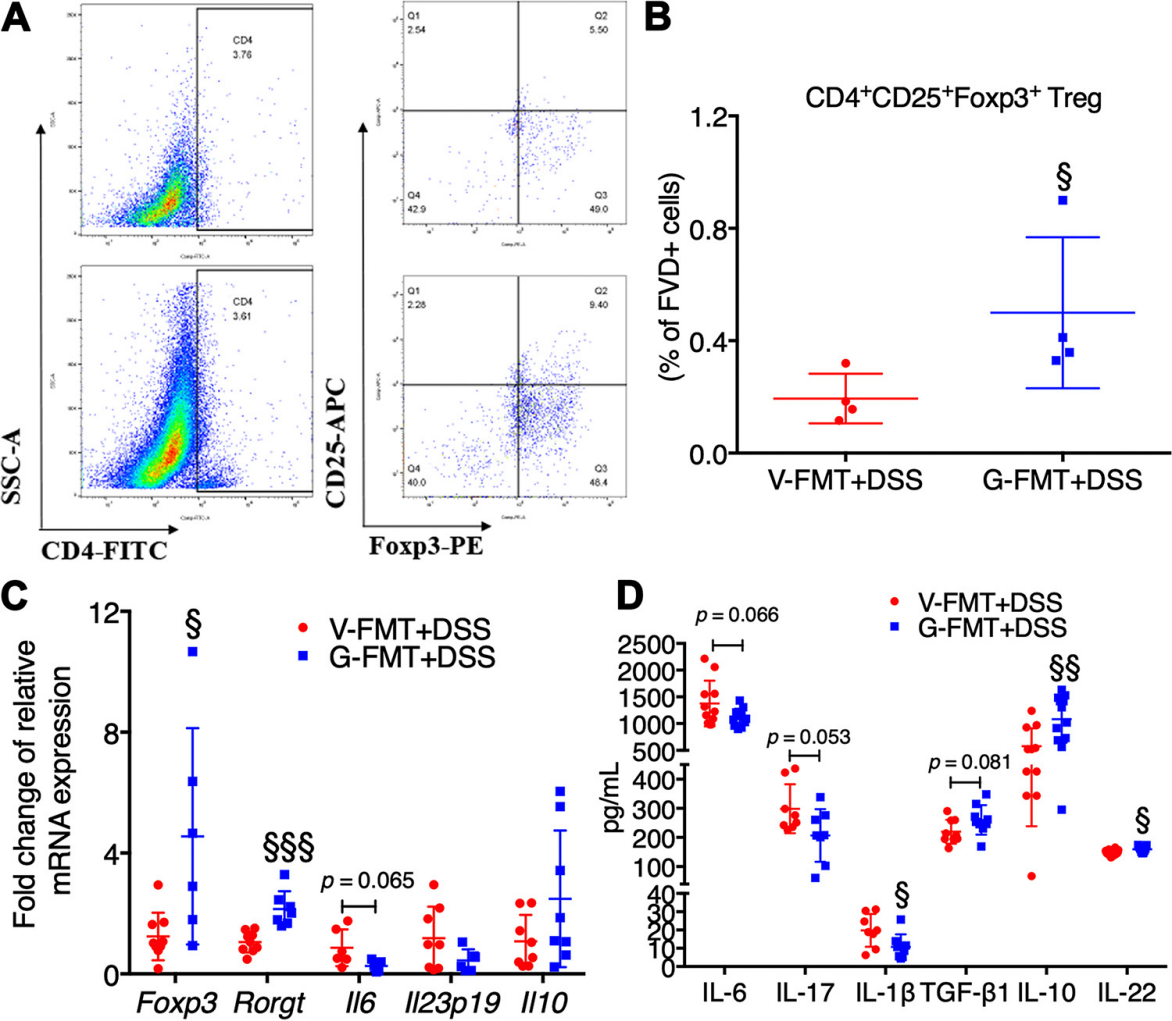
FMT from GML-treated mice promoted anti-inflammatory response upon colitis induction. (A) CD4^+^ CD25^+^ Foxp3^+^ lymphocytes of colonic lamina propria were analyzed by flow cytometry. (B) Indicated percentages are from viable cells gated on FVD (*n* = 4 mice/group). (C) Colonic mRNA levels of *Foxp3*, *Rorgt*, *Il6*, *Il23p19*, and *Il10* assessed by RT-qPCR (*n* = 6 to 8 mice/group). (D) Serum levels of IL-6, IL-17, IL-1β, TGF-β1, IL-10, and IL-22 assessed by ELISA (*n* = 9 to 12 mice/group). §, *P* < 0.05; §§, *P* < 0.01; §§§, *P* < 0.001, versus V-FMT+DSS group, determined by a two-tailed Student *t* test.

In line with the improved histological features, transplant of GML-modulated microbiota resulted in a tendency toward reduction of *Il6* in the colonic tissue (Fig. 5C). Moreover, the *Il10* level in the G-FMT+DSS group (2.486 ± 0.7987) was 2-fold higher than that in the V-FMT+DSS group (1.076 ± 0.3108), although no statistical difference was observed (Fig. 5C). However, the mRNA levels of *Il23p19*, *Tnfa*, *Il17*, *Il1b*, *Tgfb1*, and *Il22*, as well as tight junction-related genes, including *Zo1*, *Occludin*, and *Jam-1*, were similar between G-FMT+DSS and V-FMT+DSS groups (Fig. 5C and Fig. S5A to H). Of interest, FMT from GML-treated donor mice reduced the serum levels of proinflammatory IL-6 (*P* = 0.066), IL-17 (*P* = 0.053), and IL-1β (*P* < 0.05) but obviously enhanced the amounts of anti-inflammatory cytokines, including transforming growth factor β1 (TGF-β1) (*P* = 0.081), IL-10 (*P* < 0.01), and IL-22 (*P* < 0.05), compared with FMT from vehicle-treated donor mice. The concentrations of TNF-α were not significantly different between the two groups (Fig. 5D and Fig. S5I).

10.1128/mBio.02148-21.5 FIG S5 Effects of FMT on the colonic gene expression and serum protein level in antibiotic-treated mice upon colitis induction. (A to H) Colonic mRNA levels of *Zo1* (A), *Occludin* (B), *Jam-1* (C), *Il17a* (D), *Tnfa* (E), *Il1b* (F), *Il22* (G), and *Tgfb1* (H) assessed by RT-qPCR. (I) Serum protein levels of TNF-α assessed by ELISA. Values are presented as means ± SD; (A to H) *n* = 5 to 8 mice/group; (I) *n* = 13 mice/group. Download FIG S5, TIF file, 0.2 MB. Copyright © 2021 Mo et al. 2021 Mo et al. https://creativecommons.org/licenses/by/4.0/ This content is distributed under the terms of the Creative Commons Attribution 4.0 International license.

### FMT from GML-treated mice reshaped the gut microbiota in antibiotic-treated mice upon colitis induction.

**FIG 6:**
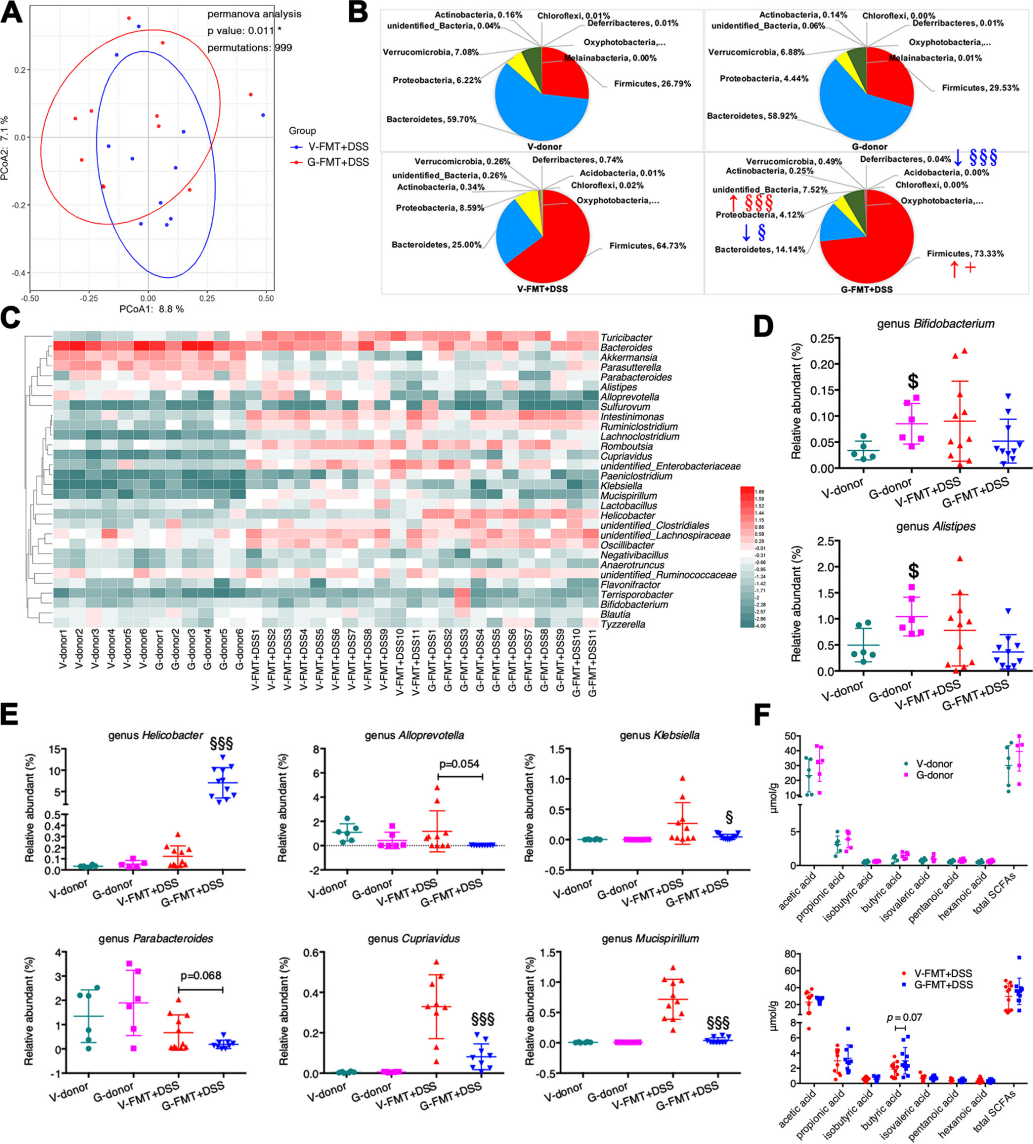
FMT of GML-treated mice reshaped the gut microbiota in antibiotic-treated mice upon colitis induction. (A) Three-dimensional (3D) PCoA plot based on OTU-level Bray-Curtis dissimilarity matrix and PERMANOVA. (B) Relative abundances of gut microbiota at the phylum level. (C) Heatmap depicting the log_10_|relative abundance| of top 30 microbial genera, when the taxa with the relative abundance as zero were expressed as −4. (D) Relative abundance of two differential microbial genera between V-donor group and G-donor group. (E) Relative abundance of six differential microbial genera between V-FMT+DSS group and G-FMT+DSS group. (F) Fecal SCFA quantification at day 15 between donor mice, as well as between V-FMT+DSS and G-FMT+DSS groups. Each dot represents an individual mouse (*n* = 6 mice/group for donor mice, *n* = 11 mice/group for recipient mice). $, *P* < 0.05, versus V-donor group. §, *P* < 0.05, and §§§, *P* < 0.001, versus V-FMT+DSS group, determined by a nonparametric factorial Kruskal-Wallis test followed by the Mann-Whitney U test when *P* was <0.05.

As shown in Fig. S6A to C, there were no differences in the microbial load and diversity between donor mice. Of importance, in line with the higher *Bifidobacterium* level in mice in the pre-GML+DSS group (Fig. 3D and E), a higher abundance of *Bifidobacterium* (*P* < 0.05) was detected in GML-treated donor mice relative to vehicle-treated mice, which further indicated that *Bifidobacterium* could be responsible for the anticolitis effect of GML pretreatment, although it was not maintained in the homologous recipient mice after FMT (Fig. 6C and D). In terms of recipient mice, we first demonstrated that antibiotic (ABX) treatment resulted in at least 10^4^-fold depletion of the gut bacterial load (Fig. S6D), significant decrease in the number of observed OTUs (Fig. S6E), and change in the microbial compositions at the phylum and genus levels (Fig. S6F and G). After FMT, the α-diversity showed no difference between the G-FMT+DSS and V-FMT+DSS groups, as reflected by Observed_species, Chao1, Shannon, and Simpson indices (Fig. S6B). Principal-coordinate analysis (PCoA) differentiated the microbiota in the G-FMT+DSS group from those in the V-FMT+DSS group, based on OTU-level Bray-Curtis dissimilarity matrix (PERMANOVA, *P* = 0.011 [Fig. 6A]), although β-diversity based on weighted and unweighted UniFrac distance showed no difference (Fig. S6C). Specifically, phylogenetic analysis indicated distinguishable microbial profiles between G-FMT+DSS and V-FMT+DSS groups. Phylum-based distinct microbial communities were explained by richer unidentified_Bacteria (*P* < 0.001), higher *Firmicutes*/*Bacteroidetes* ratio (*P* < 0.05), and reduced levels of *Proteobacteria* (*P* < 0.05) and *Deferribacteres* (*P* < 0.001) in the G-FMT+DSS group than in the V-FMT+DSS group (Fig. 6B). When looking at the genus level, *Helicobacter* (specifically species Helicobacter ganmani) mainly contributed to the increase of unidentified_Bacteria and was equally abundant in the G-FMT+DSS group (*P* < 0.001, Fig. 6C to E and Fig. S7). In contrast to *Helicobacter*, a tendency of lower *Alloprevotella* (*P* = 0.054, within *Bacteroidetes*) and significantly reduced *Mucispirillum* (*P* < 0.001, within *Deferribacteres*) and Klebsiella and *Cupriavidus* (*P* < 0.05 and *P* < 0.001, respectively, both within *Proteobacteria*) were observed in the G-FMT+DSS group compared with the V-FMT+DSS group (Fig. 6C to E). Of note, the fecal microbial communities of the recipient mice were generally distinct from those of their corresponding donor mice. These differential taxa, including the higher abundance of *Helicobacter* and lower abundance of *Alloprevotella*, *Mucispirillum*, Klebsiella, and *Cupriavidus* seen in the G-FMT+DSS group, were not observed in GML-treated donor mice. Taken together, colitis remission induced by FMT from GML-treated mice could be partly ascribed to microbial reconstruction in the recipient mice without closely resembling the microbial pattern in the donor mice. Previous studies have highlighted that the inflammation could also influence the reshaping of the gut microbiota (10); however, in the current study, GML-modulated microbiota was more resistant to DSS-induced colitis.

10.1128/mBio.02148-21.6 FIG S6 The amount and diversity of the gut microbiota between the donor mice and the recipient mice and effects of ABX treatment on the gut microbiota before FMT. (A) Feces collected from donor mice were diluted 10^4^-fold in PBS buffer and plated on Columbia blood agar base containing 5% sterile defibrinated sheep blood to culture for 3 days. (B) α-Diversity-related indices, including Observed_species, Chao1, Shannon, and Simpson indices in mice from V-donor, G-donor, V-FMT+DSS, and G-FMT+DSS groups. (C) β-Diversity based on weighted and unweighted UniFrac distance in mice from V-donor, G-donor, V-FMT+DSS, and G-FMT+DSS groups. (D) Feces collected from ABX-treated mice were diluted 10-fold in PBS buffer and plated on Columbia blood agar base containing 5% sterile defibrinated sheep blood to culture for 3 days. (E) Reduction in operational taxonomic units (OTUs) of mice after ABX treatment for 4 weeks. (F and G) Relative abundance of phyla and genera in mice after ABX treatment for 4 weeks. Download FIG S6, TIF file, 0.5 MB. Copyright © 2021 Mo et al. 2021 Mo et al. https://creativecommons.org/licenses/by/4.0/ This content is distributed under the terms of the Creative Commons Attribution 4.0 International license.

10.1128/mBio.02148-21.7 FIG S7 FMT of GML-treated mice reshaped the gut microbiota at the species level in ABX-treated mice upon colitis induction. (A) Heatmap depicting the log_10_|relative abundance| of top 25 microbial species among the four groups, when the taxa with the relative abundance as zero were expressed as −4. (B) Relative abundance of four differential microbial species between V-FMT+DSS group and G-FMT+DSS group. Each dot represents an individual mouse. Values are presented as means ± SD; *n* = 6 mice/group for donor mice, *n* = 11 mice/group for recipient mice. §, *P* < 0.05, and §§§, *P* < 0.001, versus V-FMT+DSS group. Download FIG S7, TIF file, 0.3 MB. Copyright © 2021 Mo et al. 2021 Mo et al. https://creativecommons.org/licenses/by/4.0/ This content is distributed under the terms of the Creative Commons Attribution 4.0 International license.

### FMT from GML-treated mice differentiates the microbial phylotypes, butyrate metabolism, and promoted Treg phenotype in antibiotic-treated mice upon colitis induction.

The LEfSe analysis indicated that mice receiving microbiota from GML-treated donor mice had higher levels of phylum *Firmicutes*, class *Clostridia*, order *Clostridiales*, and *Helicobacter* (from phylum to genus), whereas mice receiving microbiota from vehicle-treated mice had richer phylum *Proteobacteria*, class *Erysipelotrichia* (*Turicibacter*), and order *Enterobacteriales* (Escherichia coli) (Fig. S8). The dominance of specific members of *Clostridia* involved in the butyrate metabolism has been acknowledged (25). As expected, there was a trend toward higher butyric acid production in the G-FMT+DSS group than in the V-FMT+DSS group (*P* = 0.07), while other SCFAs showed no difference (Fig. 6F). Furthermore, epidemiological data have linked the protective benefit of Helicobacter pylori, a species of *Helicobacter*, against the relative risk of IBD (26). And Helicobacter hepaticus, another species of *Helicobacter*, specifically promoted the differentiation of colonic RORγt-expressing induced Treg (iTreg) cells, which restore eubiosis by facilitating microbiota colonization and suppressing colitis-related inflammation (27). Thus, the colonic lamina propria lymphocytes (LPLs) were analyzed with flow cytometry. Results revealed that FMT from GML-treated mice upregulated the levels of CD4^+^ CD25^+^ Foxp3^+^ Treg cells (Fig. 5A and B, *P* < 0.05), as well as dramatically increasing the transcriptional levels of *Foxp3* and *Rorgt* (Fig. 5C). Our findings were parallel to the previous data showing that the specific microbial taxa (such as *Clostridia* and *Helicobacter*), butyric acid, Tregs, and the cross talk between them may have a predominant role in preventing DSS colitis (28).

10.1128/mBio.02148-21.8 FIG S8 Key phylotypes of gut microbiota identified by LEfSe analysis with linear discriminant analysis (LDA) scores of >4 among V-donor, G-donor, V-FMT+DSS, and G-FMT+DSS groups. Download FIG S8, TIF file, 0.5 MB. Copyright © 2021 Mo et al. 2021 Mo et al. https://creativecommons.org/licenses/by/4.0/ This content is distributed under the terms of the Creative Commons Attribution 4.0 International license.

### Correlations among GML-induced gut microbiota, significantly altered colitis-related parameters, and inflammation-related factors.

**FIG 7:**
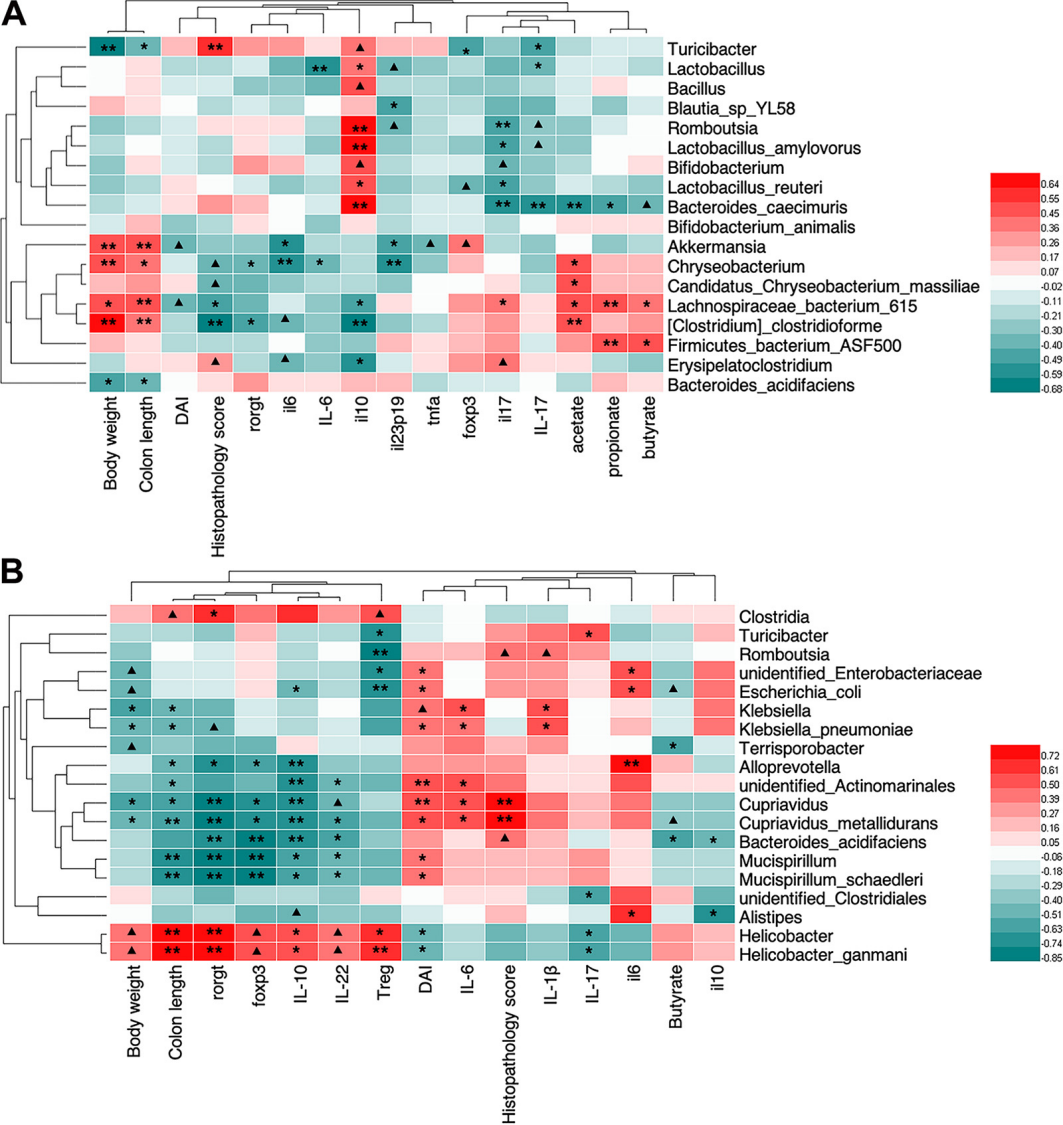
The Spearman correlation between the specific or differential microbial taxa and the colitis-related indicators among Veh, DSS, pre-GML+DSS, and co-GML+DSS groups (A) and between V-FMT+DSS and G-FMT+DSS groups (B). Red squares indicate positive correlations, and green squares indicate negative correlations. ▴, 0.05 < *P* < 0.1, means that correlations remained trending significant, and *, *P* < 0.05, and **, *P* < 0.01, mean statistically significant after correction for false-discovery rate.

We subsequently correlated significantly altered colitis-related parameters (including body weight, colon length, DAI, and histological scores) and pro-/anti-inflammatory cytokines with specific differential microbial taxa (Fig. 7). Spearman rank correlation analysis showed that the positive correlation between *Turicibacter* abundances and histological scores and the negative correlation between *Turicibacter* abundances and body weight and colon length were coincident in two independent experiments. Notably, the abundance of *Turicibacter* was remarkably increased by DSS induction but was reversed by GML pretreatment or FMT from GML-treated donor mice, suggesting that *Turicibacter* could be identified as one of the symbolic microbial taxa of DSS-induced colitis. Moreover, a significant increase of two gut commensals, *Lactobacillus* and *Bifidobacterium*, in the pre-GML+DSS group was correlated with lower levels of colonic *il6*, *il23p19*, and *il17* mRNA and lower circulating IL-6 and IL-17 levels, indicating negative correlations between *Lactobacillus* and *Bifidobacterium* and colonic and systemic inflammation (Fig. 7A). A good correlation between *Chryseobacterium* and colitis-related parameters and inflammatory status is presented in Fig. 7A. Referring to FMT assay (Fig. 7B), in accordance with the predicted capacity of *Helicobacter*, which was more abundant in the G-FMT+DSS group than the V-FMT+DSS group, to promote the differentiation of Tregs, we observed a positive correlation between *Helicobacter* (*H. ganmani*) and proportion of Foxp3^+^ Tregs (*R*^2^ = 0.709, *P* < 0.01) and *Foxp3* (*R*^2^ = 0.502, *P* = 0.0506) and *rorgt* (*R*^2^ = 0.730, *P* < 0.01). Similar correlations between *Clostridia* and Treg and *rorgt* were observed. In contrast, *Cupriavidus* (Cupriavidus metallidurans), Klebsiella (Klebsiella pneumoniae), *Mucispirillum* (Mucispirillum schaedleri), and E. coli, which were lower in the G-FMT+DSS group than the V-FMT+DSS group, were positively correlated with histological scores, DAI, and proinflammatory cytokines but negatively correlated with body weight, colon length, and anti-inflammatory factors significantly (Fig. 7B). These observations indicated that the distinct gut microbiota patterns reshaped by GML pretreatment and/or FMT trial were both linked to the moderation of colitis-related parameters and the regulated production of colonic inflammation-related factors.

## DISCUSSION

The present study demonstrated for the first time that GML can ameliorate DSS-induced colitis in C57BL/6 mice. DSS resulted in colonic inflammatory response and epithelial ulceration, while GML dramatically reversed DSS-mediated inflammation and injury. Furthermore, both DSS and GML pretreatment increased expression of colonic *il10*, indicating that the favorable effect of GML was not mediated directly via IL-10, which is crucial for preventing spontaneous colitis and maintaining homeostasis (29). Previous research also found increased expression of colonic *il10* in DSS-treated mice (30). Of interest, GML pretreatment seemed to be more effective in colitis management than GML cotreatment, although GML cotreatment exhibited a favorable effect on weight change in the early days of DSS application. One possible explanation could be that GML might have a protective effect in the early stage of inflammation. When the inflammatory response was aggravated and the mucosal layer was impaired, GML would be inadequate to rescue the disturbance and, instead, might be detrimental because of its emulsifying property. However, the intestinal permeability analysis showed no obvious difference among different groups, indicating that GML exerts its anticolitis effect in some other way. Specifically, the distinct microbial profiles and microbe-derived SCFAs between pre-GML+DSS and co-GML+DSS groups might be responsible for the case. Mice in the pre-GML+DSS group but not the co-GML+DSS group contained a lower abundance of inflammation-related *Turicibacter* and higher proportions of *Bifidobacterium* and *Lactobacillus*, which ae well-known beneficial SCFA-producing microbes and have anti-inflammatory and anti-colorectal cancer effects in humans and mice (31, 32). Similarly, a previous study demonstrated that prebiotic inulin supplementation promoted indigenous *Bifidobacterium* and *Lactobacillus*, which were associated with the decreased risk of IBD development (33). Similarly to IBD patients who have typically decreased microbial diversity and attenuated ability to produce SCFAs (2), DSS resulted in significant decline in the microbiota diversity and total SCFAs, especially acetic acid and butyric acid. In contrast, GML, regardless of pretreatment or cotreatment, reversed the DSS-induced dysbiosis in microbial diversity. Accordingly, butyric acid and propionic acid, which were marginally restored in the pre-GML+DSS group relative to the DSS group, could contribute to ameliorating experimental colitis (28). The aforementioned results were consistent with the previous reports that *Bifidobacterium* has been regarded as an acetate-to-butyrate converting organism, and the synthetic butyrate, in turn, promoted the growth of *Bifidobacterium* and *Lactobacillus* in the gut (34, 35). It has been verified that *Bifidobacterium* and butyrate have beneficial effects on ameliorating mucosal inflammation and related symptomology (8, 31, 32). Notably, the higher *Bifidobacterium* in the pre-GML+DSS group was in line with the higher *Bifidobacterium* in GML-treated donor mice, indicating that GML-modulated specific microbial taxa were responsible for its ant-inflammatory potential. Indeed, FMT from GML-treated mice has an advantage over that from vehicle-treated mice in achieving colitis remission, and in shifting the microbial communities and immune response toward better homeostasis. In contrast, different gut microbiota patterns were involved in the GML prevention experiment and FMT trial.

Similarly to the previous FMT trials (36, 37), the variations of gut microbiota in the recipient mice explain the distinct effects on colitis, which further provided a perspective to identify novel microbial members that are responsible for the anticolitis effect. Mice receiving microbiota from vehicle-treated mice were associated with dominant *Erysipelotrichaceae* (*Turicibacter*) and *Enterobacteriaceae* (E. coli) and significantly increased *Alloprevotella*, *Mucispirillum*, Klebsiella, and *Cupriavidus*, which have been shown to facilitate intestinal inflammation or colorectal cancer (38, 39). LEfSe analysis of DSS and V-FMT+DSS groups highlighted that *Erysipelotrichaceae* (*Turicibacter*) and *Enterobacteriaceae* (E. coli) could be deemed the indicator phylotypes of active colitis. Consistently, similar change in *Erysipelotrichaceae* has been described in IBD patients and inflammation-related mouse models (40), and *Enterobacteriaceae* (E. coli) could be selectively enhanced by inflammatory response (41). In contrast, FMT from GML-treated mice during colitis resulted in a trend of increase of *Firmicutes* (represented by *Clostridia*) and a marked decrease of *Proteobacteria* (represented by Klebsiella, *Cupriavidus*, and *Enterobacteriaceae*), in parallel with clinical remission by FMT or other intervention trials on mouse colitis model (42). The previous results implied that members of *Clostridia* were protective against DSS-induced colitis by restoring butyric acid and colonic Foxp3^+^ Tregs (25) and could be used as a potential biomarker to guide therapy (39, 43). As expected, transplant of GML-mediated microbiota promoted butyric acid concentration, shaped Foxp3^+^ Treg differentiation in the LPLs, and shifted cytokine profiles at the gene and protein levels, including IL-10 and IL-6, which may in part be responsible for the significant amelioration of colitis-related status and symptoms caused by DSS. Moreover, IL-6 plays a vital role as a signal marker in restraining Treg maturation and triggering Th17-mediated inflammation (44). Thus, the marked reduction of IL-6 further supported the conclusion that GML-altered microbiota maintains Treg/Th17 balance upon DSS induction. Of importance, genus *Helicobacter* (specifically *H. ganmani*), dominant in the G-FMT+DSS group compared with the V-FMT+DSS group, was nonpathogenic in mice and humans and uncorrelated with colitis (45). Previous observations reported that treatment with Tregs resulted in colitis remission in TRUC mice by preferentially facilitating the proliferation of *H. ganmani* (46). Here, the augment of *Helicobacter* and *H. ganmani* may contribute to the induction of RORγt^+^ Foxp3^+^ Treg cells, both of which were responsible for the mechanism whereby FMT from GML-treated mice achieved a faster remission of DSS-induced colitis. Moreover, the positive correlation between *H*. *ganmani* and RORγt^+^ Foxp3^+^ iTregs fit in exactly with the prospect raised previously that the homeostasis in IBD patients could be reestablished by exploring or managing nonpathogenic microorganisms to induce iTreg responses (27, 47). The current experimental data motivated several trials of *H. ganmani* as IBD therapy.

In conclusion, our findings revealed that GML-modulated microbiota, whether retained within the conventional mice or moved by FMT into the recipient mice, were similarly capable of preventing DSS-induced colitis, despite the fact that distinct gut microbiota patterns were involved in these two conditions. As has been demonstrated recently, certain amounts of clades from different taxa may share similar functions, such as SCFA metabolism and inflammation suppression, because of the functional redundancy of the microbial community (40, 48, 49). For example, *Bifidobacterium* enriched in the pre-GML+DSS group and GML-treated donor mice and *Helicobacter* enriched in the G-FMT+DSS group can potentially promote colonic indoleamine 2,3-dioxygenase production and Treg differentiation, both of which play vital roles in attenuating inflammation (50, 51).

## MATERIALS AND METHODS

### Animals, colitis induction, and GML treatment.

Male C57BL/6 mice (4 to 5 weeks old) were purchased from the SLAC Laboratory Animal Co. Ltd. (Shanghai, China) and housed under specific-pathogen-free conditions with food and water *ad libitum*. Food-grade glycerol monolaurate (GML) was obtained from Kangyuan Food Science and Technology Co., Ltd. (Hangzhou, China). DSS (dextran sodium sulfate; molecular weight [MW] 36,000 to 50,000; MP Biomedicals, USA) was dissolved in the drinking water to a concentration of 2.5%. Mice were randomized to four groups (*n* = 15/group) as follows: Veh group, mice received vehicle (days 0 to 28); DSS model group, vehicle (days 0 to 28) and DSS application on days 21 to 28; GML pretreatment group (pre-GML+DSS), GML (days 0 to 21), vehicle, and DSS (days 21 to 28); GML cotreatment group (co-GML+DSS), GML (days 0 to 28) and DSS added (days 21 to 28). Vehicle (50% polyethylene glycol 400 and 50% distilled water) and GML (6 mg/mouse/day, dissolved in vehicle) were administered daily by gavage (300 μl/mouse). Weight of mice was recorded daily during DSS induction and expressed as a percentage of the weight at day 21 (at which time DSS was applied) defined as 100%. Fresh feces were harvested at day 4 after DSS application. At day 28, mice were euthanized (Fig. 1A). Blood was collected, and serum was generated by centrifugation (3,000 × *g* for 20 min at 4°C). Mice were euthanized, and colon was quickly removed for length measurement and collected for further assessment. All animal experiments were in accordance with federal guidelines and approved by the Institutional Animal Care and Use Committee of Zhejiang Chinese Medical University (China, approval no. IACUC-20180319-04).

### Evaluation of the DAI.

All mice were assessed for disease activity index (DAI) daily which was rated from 0 to 4 by scoring weight loss, stool consistency, and bloody stool as previously described (52). The DAI was calculated as the average of the total scores: weight loss (0, none; 1, 1 to 5%; 2, 5 to 10%; 3, 10 to 15%; 4, >15%), stool consistency (0, none; 2, loose stools; 4, diarrhea), and bloody stool (0, normal; 1, occult blood not visible to the naked eye; 2, slight bleeding; 3, moderate bleeding; 4, gross bleeding).

### Histological assessment.

Colonic samples were fixed overnight in 10% formalin, dehydrated, and embedded into paraffin according to a standard procedure. The paraffin sections of colonic samples were subjected to hematoxylin and eosin (H&E) staining at core facilities, Zhejiang University School of Medicine (Hangzhou, China). The extent of colitis was assessed according to the previously described protocols (30). The following parameters were examined on a scale of 0 to 4, respectively: (i) epithelial damage—0, normal appearance; 1, mild abnormality; 2, probable erosion or multifocal abnormalities; 3, unequivocal erosion or multifocal abnormalities; 4, ulcer or severe diffusion; (ii) chronic inflammatory infiltrate—0, no infiltration; 1, mild but unequivocal infiltrate; 2, infiltrate, moderate increase; 3, infiltrate, marked increase; 4, accumulation in lumen; (iii) extent of inflammation—0, none; 1, mild diffusion around the crypt area; 2, moderate diffusion in the mucosa; 3, diffusion in submucosa; 4, transmural diffusion, pronounced edema. Histopathology scoring was calculated in a blind manner for a combined score of 0 to 20.

### *In vivo* intestinal permeability assay.

Mice were fasted for 4 h and administered FITC-dextran (4 kDa, 12 mg/mouse; Sigma-Aldrich) by gavage. Serum was collected 3 h later, and median fluorescence intensity (MFI) was quantified by a multiplate reader (485/535 nm).

### Antibiotic-treated mice and fecal microbiota transplantation (FMT).

To deplete the gut microbiota, mice were treated with antibiotic (ABX)-containing water, including ampicillin (1 g liter^−1^), neomycin (1 g liter^−1^), metronidazole (1 g liter^−1^), and vancomycin (0.5 g liter^−1^) for 4 weeks (53). Microbiota depletion was confirmed by anaerobic culture of 10-fold-diluted fecal suspension on Columbia blood agar base containing 5% sterile defibrinated sheep blood. The littermates administered vehicle or GML intragastrically for 4 weeks were used as donor mice and divided into the V-donor and G-donor groups, respectively. Fresh feces collected from each group were pooled and homogenized in the sterile phosphate-buffered saline (PBS), respectively. The fecal supernatant obtained by centrifugation was orally inoculated into ABX-treated recipient mice, respectively (250 μl/mouse, daily), for 3 days, whereupon ABX-treated recipient mice consumed 2.5% DSS-containing water to induce colitis (Fig. 4A). Correspondingly, the recipient mice were divided into V-FMT+DSS and G-FMT+DSS groups, respectively. Feces and serum were collected. Mice were sacrificed at day 15, and colon was quickly collected for length measurement and further assessment.

### Quantification of mRNAs by RT-qPCR.

Total RNA from colonic samples was extracted using the FastPure Cell/Tissue total RNA isolation kit (Vazyme Biotech Co., Ltd.). The cDNA generated by HiScript II Q RT SuperMix for qPCR (+gDNA wiper) (Vazyme Biotech Co., Ltd.) was analyzed using primers for the indicated genes. Real-time quantitative PCR (RT-qPCR) was performed with 2×ChamQ SYBR Color qPCR Master Mix (Vazyme Biotech Co., Ltd.) and run in a LightCycler 480 system (Roche) according to the manufacturer’s instructions. Genes *Foxp3*, *Il6*, *Tnfa*, *Il23p19*, *Il10*, *Il17a*, *Il22*, *Il1b*, *Tgfb1*, *Rorgt*, *Zo1*, *Occludin*, and *Jam-1* were evaluated, and *β-actin* was used as the housekeeping gene for colonic tissue. Relative expression of mRNA levels was quantified using the threshold cycle (2^−ΔΔ^*^CT^*) method as described previously (54). The primers are shown in Table 1.

**TABLE 1:** Primer sequences used in the qRT-PCR assays

Gene	Sequence (5′–3′)
*Zo1* Forward	AGGACACCAAAGCATGTGAG
*Zo1* Reverse	GGCATTCCTGCTGGTTACA
*Occludin* Forward	AGGAGTTAACGTCGTGGACCGG
*Occludin* Reverse	GGCAGGAATGCTGTCATTTGCAG
*Jam-1* Forward	CCCCGAGTGGAGTGGAAGTTCG
*Jam-1* Reverse	GAGGTCTGTTTGAATTCCCCCTC
*Foxp3* Forward	GAGAAAGCGGATACCAAA
*Foxp3* Reverse	TGTGAGGACTACCGAGCC
*Il6* Forward	TCACAGAAGGAGTGGCTAAG
*Il6* Reverse	ACTAGGTTTACCGAGTAGAT
*Tnfa* Forward	GGGGTGGAAAGTGAATACTA
*Tnfa* Reverse	GCAGAGTGGTTGTAATGGTT
*Il23p19* Forward	AGCGGGACATATGAATCTACTAAGAGA
*Il23p19* Reverse	GTCCTAGTAGGGAGGTGTGAAGTTG
*Il10* Forward	GACCAGCTGGACAACATACTGCTAA
*Il10* Reverse	GATAAGGCTTGGCAACCCAAGTAA
*Tgfb1* Forward	TGACGTCACTGGAGTTGTACGG
*Tgfb1* Reverse	GGTTCATGTCATGGATGGTGC
*Rorgt* Forward	TGCAAGACTCATCGACAAGG
*Rorgt* Reverse	AGGGGATTCAACATCAGTGC
*Il17a* Forward	TCTCTGATGCTGTTGCTGCT
*Il17a* Reverse	CGTGGAACGGTTGAGGTAGT
*Il22* Forward	TCCAACTTCCAGCAGCCATACATC
*Il22* Reverse	GCACTGATCCTTAGCACTGACTCC
*Il1b* Forward	TGCCACCTTTTGACAGTGATG
*Il1b* Reverse	AAGGTCCACGGGAAAGACAC
*β-actin* Forward	GTGCTATGTTGCTCTAGACTTCG
*β-actin* Reverse	ATGCCACAGGATTCCATACC

### Enzyme-linked immunosorbent assay.

The inflammatory state of serum was assessed through measurement of LPS (Wuhan Colorfulgene Biological Technology Co., Ltd., China) and cytokines, including IL-6, TNF-α, IL-17, IL-1β, IL-10, IL-22, and TGF-β (eBioscience, San Diego, CA, USA), according to the instructions of the manufacturer.

### Gut microbiota analysis.

Fecal bacterial DNA was extracted with the QIAamp DNA stool minikit (Qiagen, Venlo, Netherlands) based on the manufacturer’s instructions. The concentration and quality of the resulting DNA were determined with a UV-visible (UV-Vis) spectrophotometer (NanoDrop 2000c; Thermo Scientific). 16S rRNA amplicon sequencing was performed at the Novogene Bioinformatics Institute (Tianjin, China) with an Illumina IonS5 XL platform. The V3-V4 hypervariable regions in the bacterial DNA were PCR amplified from each sample with a primer set (F341, 5′-ACTCCTACGGGRSGCAGCAG-3′, and R806, 5′-GGACTACVVGGGTATCTAATC-3′) containing a unique barcode, which was used to tag PCR products from respective samples (55). The resulting sequences were analyzed according to previous work (53, 56). In detail, the resulting sequencing reads were demultiplexed and quality filtered by the following procedures: (i) reads that had more than three consecutive low-quality base calls were discarded using Cutadapt v1.9.1 and were assigned to respective sample according to the unique barcodes, (ii) adaptors and barcodes were trimmed and reads with average quality below Q20 were removed, and (iii) chimeras were dumped using the Userch algorithm (57, 58). The processed sequences with a 97% pairwise identity threshold were clustered into the same operational taxonomic units (OTUs) using Uparse v7.0.1001 (59) and classified taxonomically using the SILVA 132 database (60). α-Diversity was estimated using the Chao1, Shannon, Simpson, and Observed_species indices and was analyzed using Quantitative Insights into Microbial Ecology software (QIIME v1.9.1). β-Diversity was evaluated using principal-coordinate analysis (PCoA) plots based on weighted UniFrac and the Bray-Curtis distance (61). To determine phylum, class, order, family, genus, and species OTUs, data were evaluated using a permutational multivariate analysis of variance (PERMANOVA) for analysis of Bray-Curtis indices. Linear discriminant analysis (LDA) effect size (LEfSe) algorithm was applied to identify specific taxa that responded to different treatments (53). Statistical analyses were performed with R (version 2.15.3).

### Measurements of fecal short-chain fatty acid (SCFA) concentrations.

Fecal samples were used to measure SCFA concentrations via gas chromatographic analysis as previously described (62). In detail, fecal pellets from single mice were weighed and homogenized in ultrapure water. The pH of fecal suspension was adjusted to 2 to 3 by adding 5 M HCl with intermittent vortexing within 15 min. The clear supernatant was collected after centrifugation for 20 min at 3,000 × *g*. 2-Ethylbutyric acid (TEBA), used as the internal standard, was added into the resulting supernatant at a final concentration of 1 mmol liter^−1^. A capillary column (30 m, 0.53 mm, 0.50 μm) with a free fatty acid phase (DB-FFAP 125-3237; J&W Scientific, Agilent Technologies Inc.) and the Shimadzu GC-2014 system were applied for gas chromatographic analysis (22).

### LPL isolation.

For evaluation of regulatory T cells (Tregs), lamina propria lymphocytes (LPLs) in the colonic samples were isolated as described previously (30). In brief, Peyer’s patches were discarded and the whole colon was opened longitudinally, minced into 0.5-cm pieces, and digested in EDTA-containing Hanks’ balanced salt solution (HBSS; 0.5 mol liter^−1^ EDTA) for 30 min at 37°C with rigorous shaking to remove intraepithelial lymphocytes and epithelial cells. Remaining tissue pieces were digested twice for 15 min in complete medium (Dulbecco’s Modified Eagle Medium [DMEM], 10% fetal calf serum [FCS]) supplemented with 1 mol liter^−1^ HEPES buffer, 145 mg ml^−1^ collagenase II (Worthington Biochemical), and 50 mg ml^−1^ DNase I (Sigma-Aldrich) with pipetting using a 1-ml syringe. The resulting cell suspensions were filtered through a 70-μm cell strainer and centrifuged for 5 min at 1,000 × *g*. Cell pellets were layered onto a 40:80% Percoll gradient and centrifuged at 2,000 × *g* at room temperature for 20 min in a Thermo Sorvall ST16R centrifuge. The LPLs collected from the 40:80% interface were resuspended in PBS at a density of 1 × 10^6^ prior to staining for flow cytometry.

### Flow cytometry.

The surface staining followed routine procedures (8, 30), and the intracellular staining was performed by use of the Foxp3 staining kit (eBioscience), according to the manufacturer’s instructions. LPL suspensions were stained for surface molecules by incubating with anti-mouse CD4-FITC (eBioscience), anti-mouse CD25-allophycocyanin (APC) (eBioscience), and rat IgG2a isotype control-phycoerythrin (PE) (eBioscience) on ice for 20 min and then washed with PBS buffer. Fixable viability dye (FVD)-eFluor 450 (eBioscience) was used to exclude dead cells. Cells were then fixed in fixation and permeabilization working solution for 30 min, followed by washing and resuspension in permeabilization buffer prior to staining with anti-mouse Foxp3-PE antibody for at least 30 min on ice. Flow cytometric analysis was performed on an LSR Fortessa analyzer (BD Biosciences), and data were analyzed using FlowJo software (Tree Star).

### Transcriptional profiles in the whole-colon tissue.

Total RNA extracted from the colonic tissue and with an integrity number larger than 7 was subjected to RNA sequencing, which was performed by the Novogene Bioinformatics Institute (Tianjin, China) with the Illumina HiSeq platform (55). Briefly, raw reads were processed through Trimmomatic v0.3.0 to remove reads containing adaptors or poly-N and reads with low quality. The resulting clean reads were aligned to mouse genome (mm9), and the reads mapped to genomic features were counted by HISAT2 v2.0.1. The differential expression of genes and the KEGG pathway were analyzed by using HTSeq v0.6.0 and clusterProfiler R package, respectively.

### Statistical analysis.

Data were expressed as means with standard deviation (SD). The lowercase letter ‘n’ means the number of mice. Data sets among Veh, DSS, pre-GML+DSS, and co-GML+DSS groups were evaluated for whether they followed the normal distribution by the Kolmogorov-Smirnov test. The result showed that the data fitted the normal distribution. The Levene test of homogeneity of variance was further performed. When the data fitted the homogeneity of variance, one-way ANOVA followed by Bonferroni’s *post hoc* test was performed. Nonparametric data among the four groups were analyzed with the Kruskal-Wallis test followed by the Mann-Whitney U test when *P* was <0.05. A two-tailed Student *t* test was applied to compare V-donor group with G-donor group or V-FMT+DSS group with G-FMT+DSS group. IBM SPSS 21.0 (Chicago, IL, USA) and GraphPad Prism 6.0 (San Diego, CA, USA) were used to perform statistical analysis. The outliers were detected and eliminated with the ROUT method (*Q* = 1%) using GraphPad Prism 6.0. Differences were considered to be statistically significant when *P* was <0.05.

### Data availability.

All the relevant data which support our findings are available from the authors on reasonable request; some have already been included in the paper and the supplemental material. Raw sequence data of microbiota which support the findings in our study have been deposited into NCBI’s Sequence Read Archive under accession number PRJNA766304. Raw sequence data of transcriptome that support our findings have been deposited in NCBI’s Sequence Read Archive under accession number PRJNA766430.
