# Supplementary figures and images for: Novel Gut Microbiota Patterns Involved in the Attenuation of Dextran Sodium Sulfate-Induced Mouse Colitis Mediated by Glycerol Monolaurate via Inducing Anti-inflammatory Responses
Source: mBio. 2021 Oct 12;12(5):e02148-21. doi: 10.1128/mBio.02148-21 (PMC8510546; doi:10.1128/mBio.02148-21)

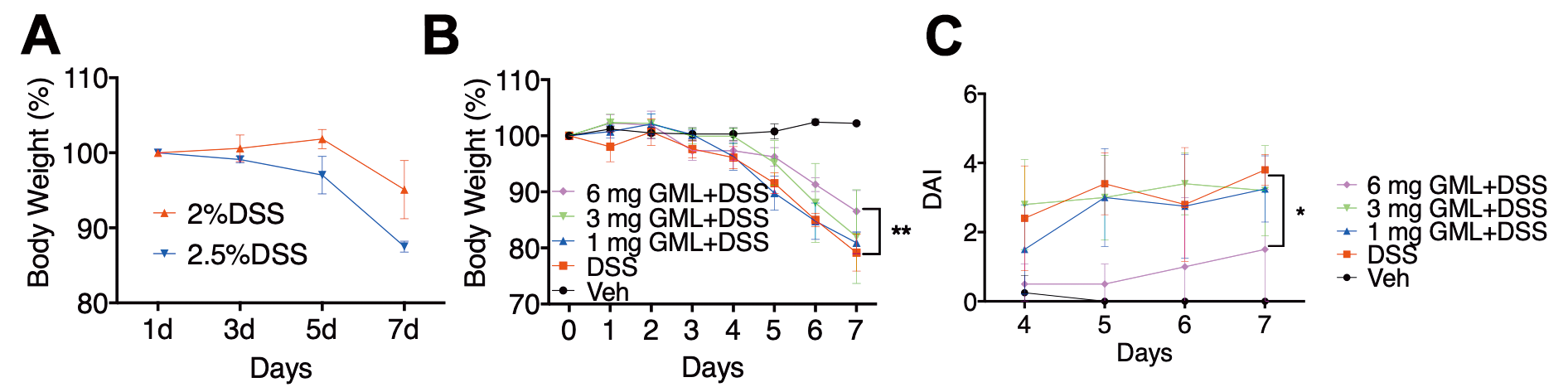

Supplement: FIG S1 [file mbio.02148-21-sf001.tif]

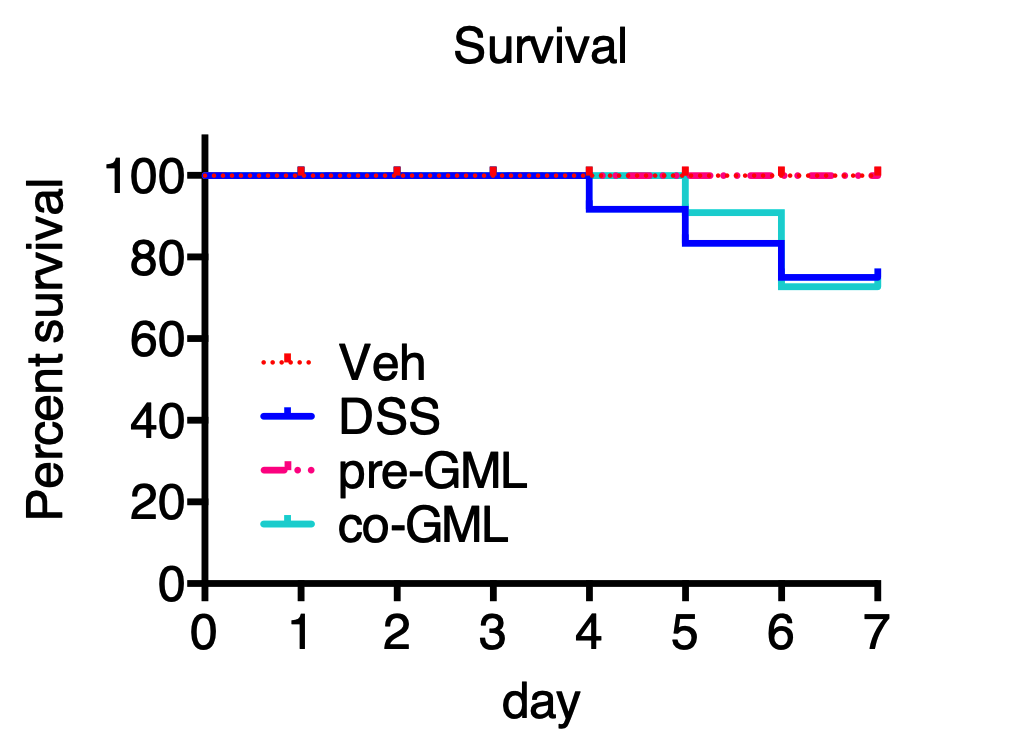

Supplement: FIG S2 [file mbio.02148-21-sf002.tif]

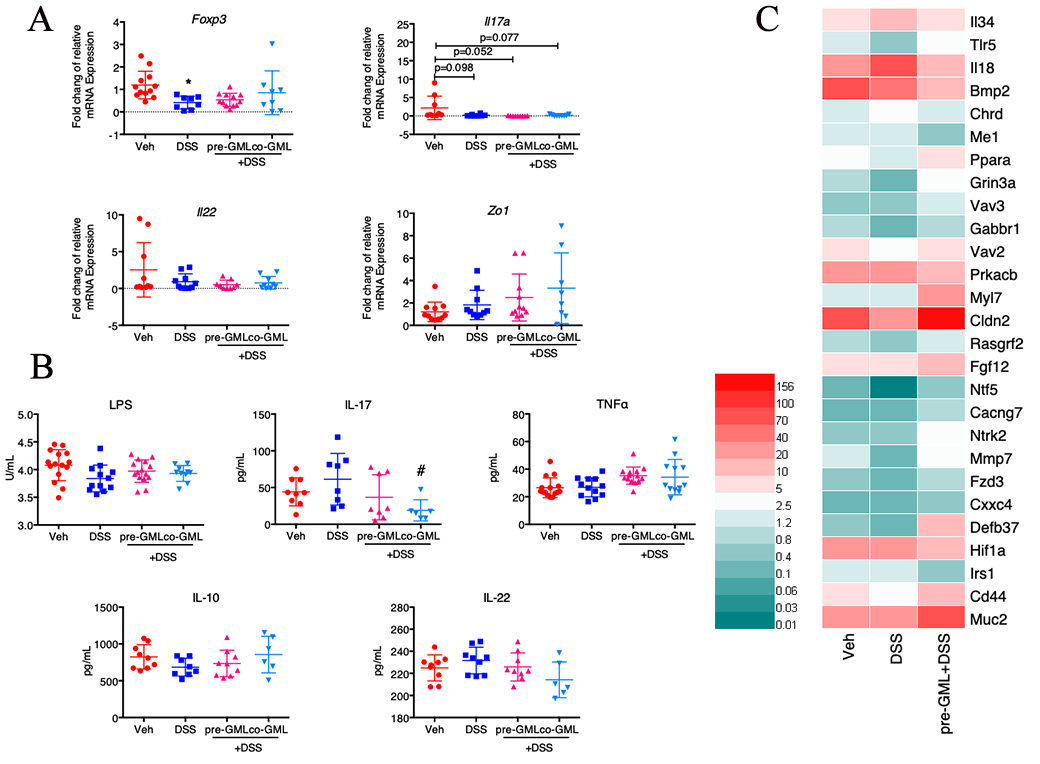

Supplement: FIG S3 [file mbio.02148-21-sf003.tif]

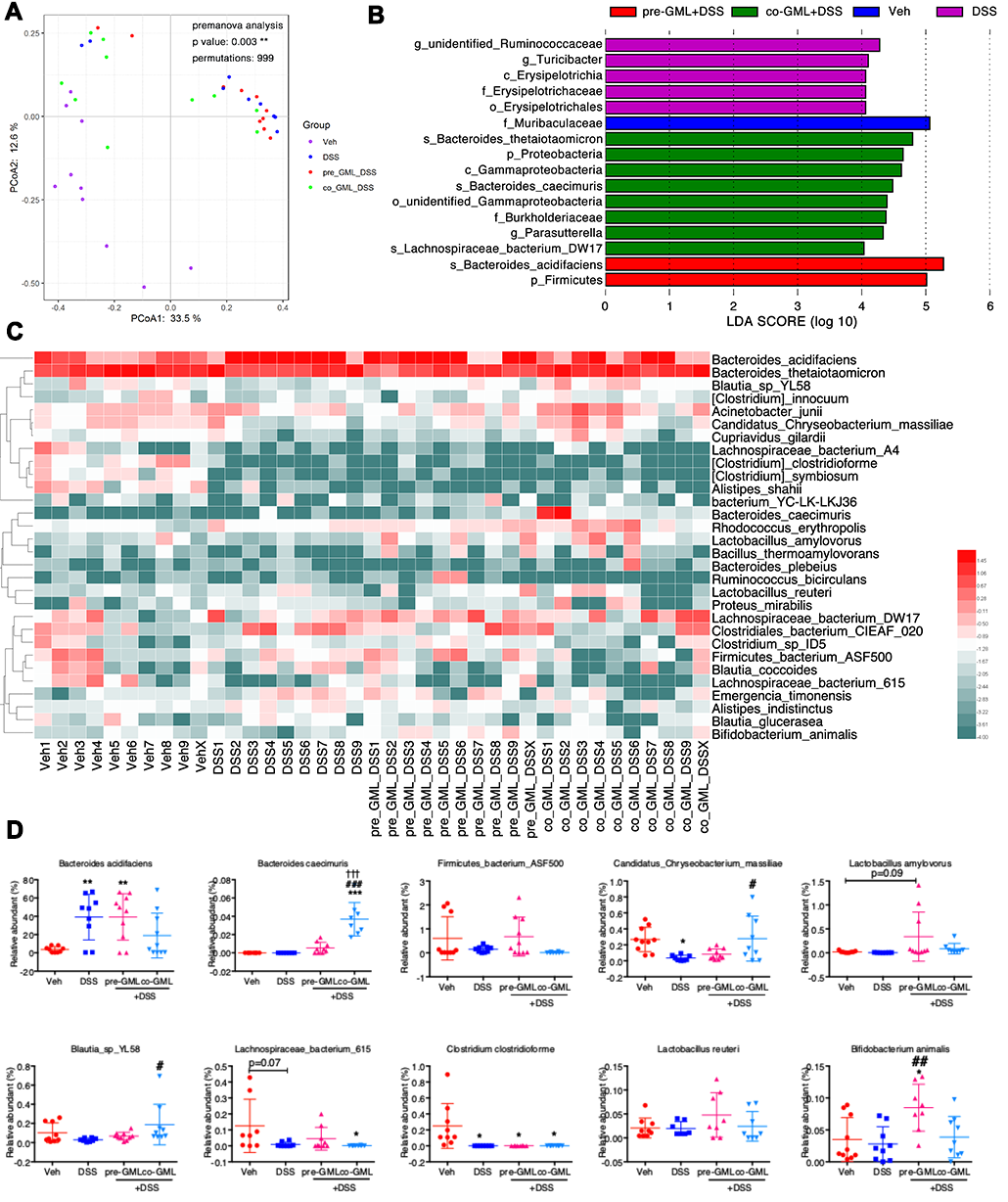

Supplement: FIG S4 [file mbio.02148-21-sf004.tif]

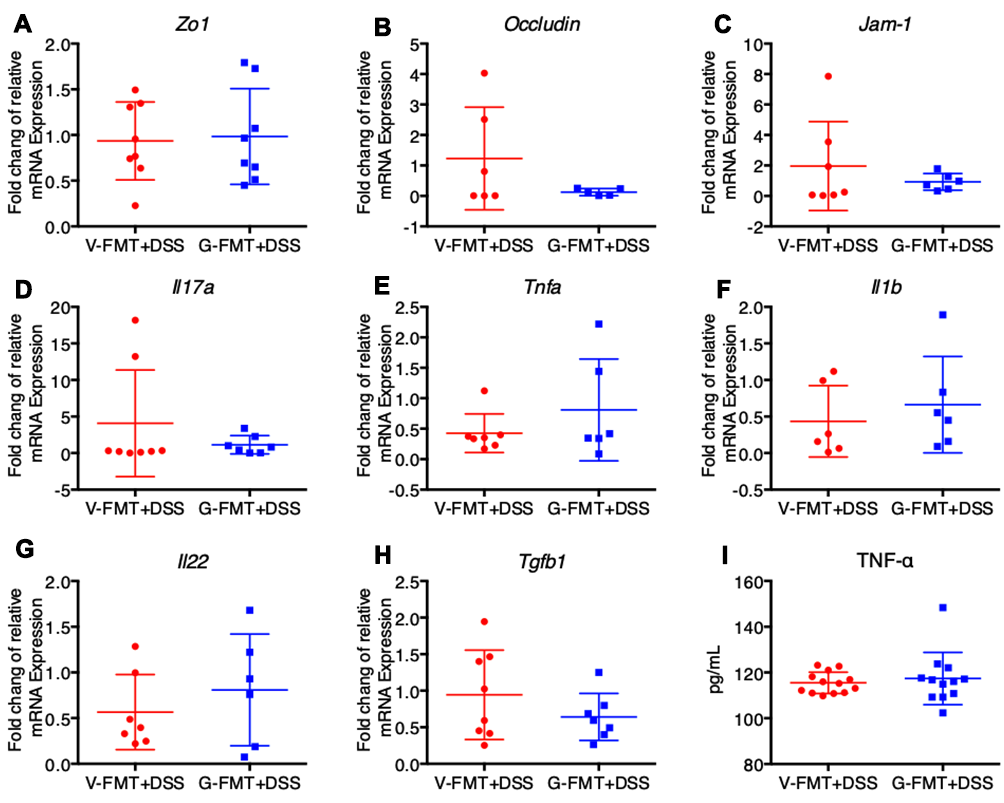

Supplement: FIG S5 [file mbio.02148-21-sf005.tif]

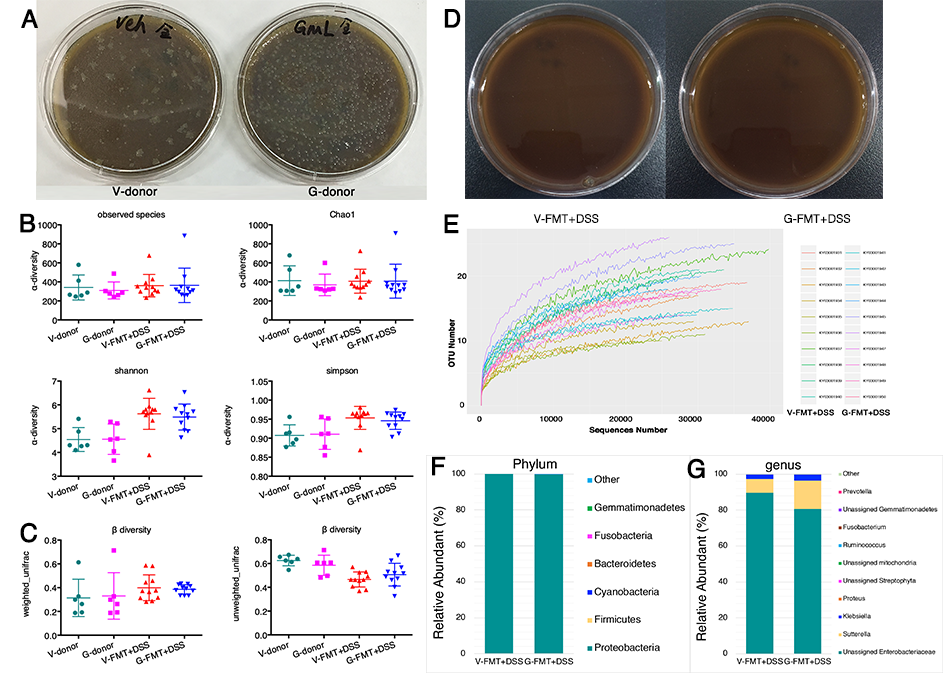

Supplement: FIG S6 [file mbio.02148-21-sf006.tif]

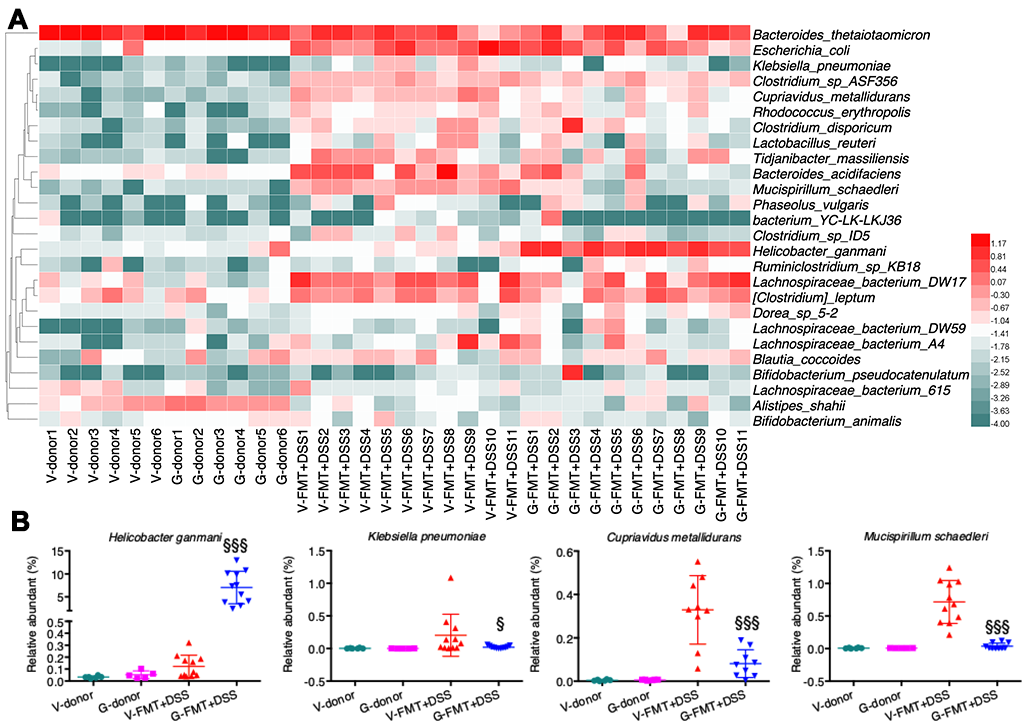

Supplement: FIG S7 [file mbio.02148-21-sf007.tif]

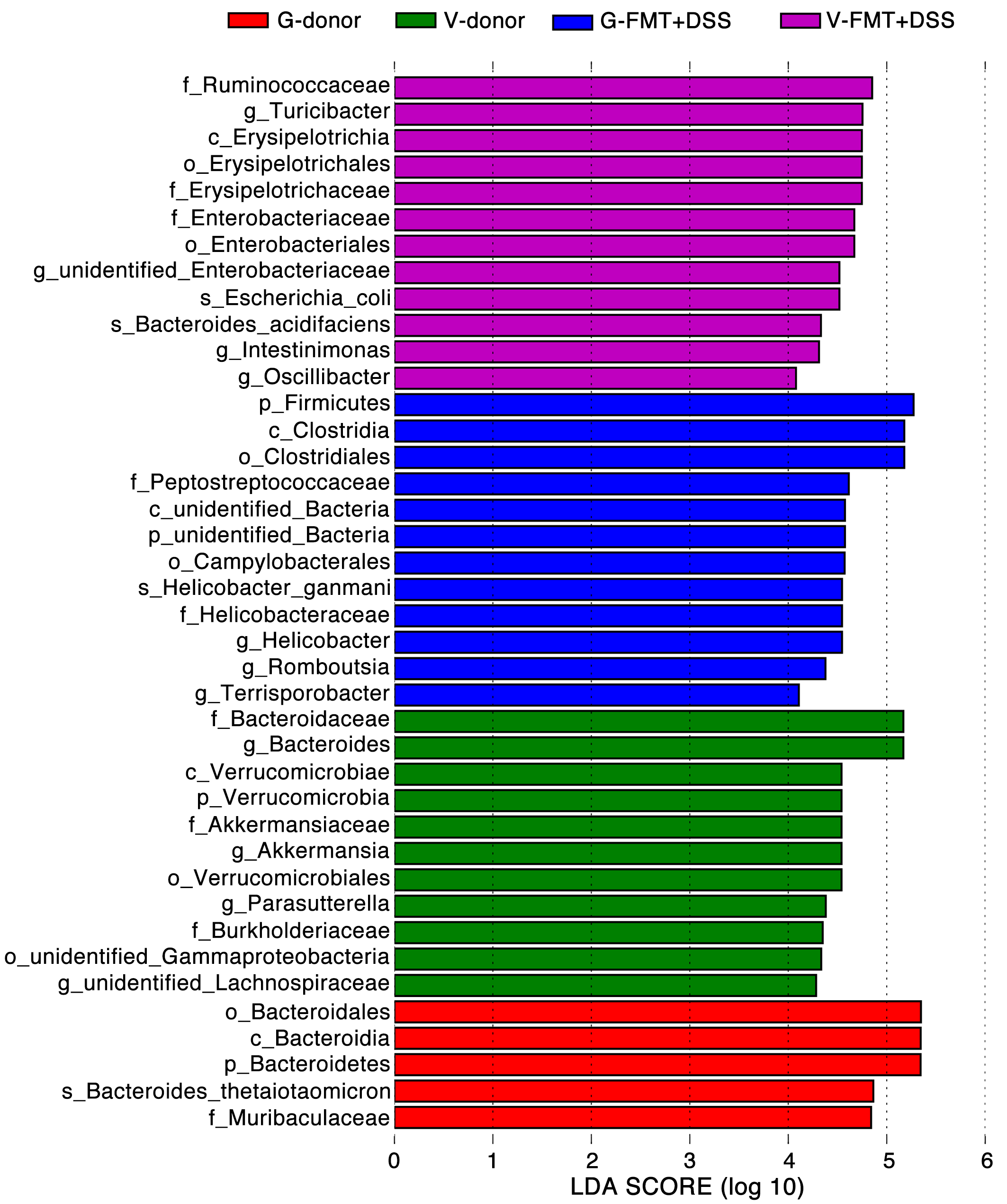

Supplement: FIG S8 [file mbio.02148-21-sf008.tif]
